# Redeployment of odontode gene regulatory network underlies dermal denticle formation and evolution in suckermouth armored catfish

**DOI:** 10.1038/s41598-022-10222-y

**Published:** 2022-04-13

**Authors:** Shunsuke Mori, Tetsuya Nakamura

**Affiliations:** Department of Genetics, Rutgers the State University of New Jersey, Piscataway, NJ 08854 USA

**Keywords:** Bone development, Experimental organisms, Developmental biology, Evolution, Evolutionary developmental biology

## Abstract

Odontodes, i.e., teeth and tooth-like structures, consist of a pulp cavity and dentin covered by a mineralized cap. These structures first appeared on the outer surface of vertebrate ancestors and were repeatedly lost and gained across vertebrate clades; yet, the underlying genetic mechanisms and trajectories of this recurrent evolution remain long-standing mysteries. Here, we established suckermouth armored catfish (*Ancistrus sp.*; Loricariidae), which have reacquired dermal odontodes (dermal denticles) all over most of their body surface, as an experimental model animal amenable to genetic manipulation for studying odontode development. Our histological analysis showed that suckermouth armored catfish develop dermal denticles through the previously defined odontode developmental stages. De novo transcriptomic profiling identified the conserved odontode genetic regulatory network (oGRN) as well as expression of *paired like homeodomain 2* (*pitx2*), previously known as an early regulator of oGRN in teeth but not in other dermal odontodes, in developing dermal denticles. The early onset of *pitx2* expression in cranial dermal denticle placodes implies its function as one of the inducing factors of the cranial dermal denticles. By comprehensively identifying the genetic program for dermal odontode development in suckermouth armored catfish, this work illuminates how dermal odontodes might have evolved and diverged in distinct teleost lineages via redeployment of oGRN.

## Introduction

Exoskeletal armor is a prime protective adaptation for thriving in a natural environment, and dermal odontodes are the most common exoskeletal feature found across the vertebrate phylogeny of extinct and extant fish. Dermal odontodes are constructed from various types of dentin sometimes covered by enamel/enameloid and primarily function as protectants and ornaments on the vertebrate body^1^. They form body armor in concert with underlying dermal bony plates in odontode-bearing fish, excepting modern cartilaginous fish^2^.

During vertebrate evolution, the first dermal odontodes shielding the body surface appeared in Ordovician (~ 460 million years ago) in pteraspidomorphi, extinct jawless fish group including anaspids or heterostracans^3^. Thereafter, throughout the evolution of jawless and jawed fish, the architecture of odontodes with underlying dermal bony plates has been continuously modified and displays morphological variability^4^. Odontodes may have been recruited into the oral cavity and transformed into teeth in the “outside-in” model during the origin of jawed fish^5^. However, due to gaps in the fossil record, the evolutionary origin of teeth continues to be disputed and revisited^6–8^.

**Figure 1:**
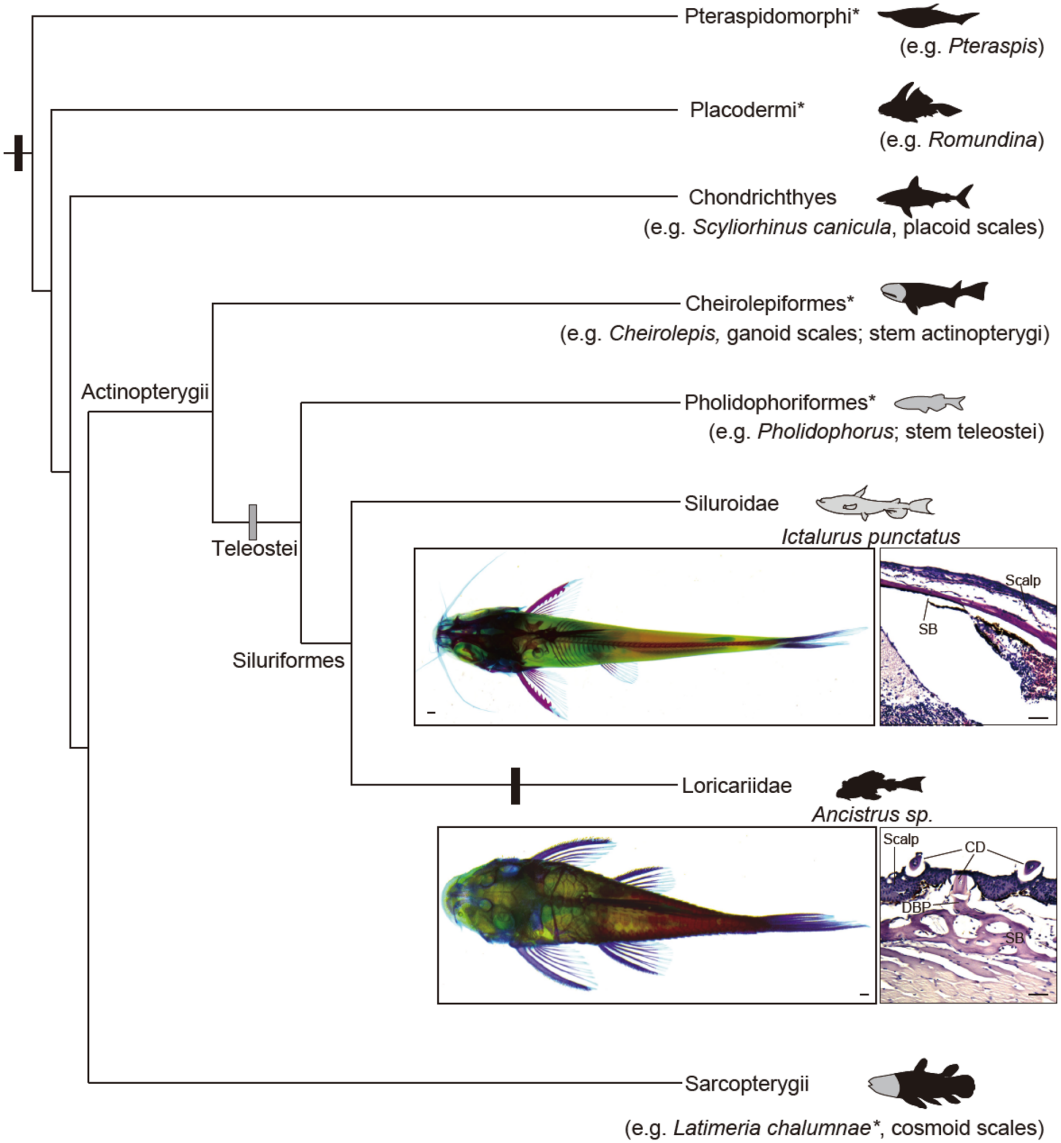
Phylogenetic distribution of dermal odontodes in vertebrates. (**A**) Simplified cladogram of the distribution of dermal odontode in vertebrates evolution^3,4,9,28^. Asterisk indicates the extinct lineages. Dermal odontodes (black) ornament whole body surface of some extinct jawless (Pteraspidomorphi) and jawed fish (placodermi), and chondrichthyans. The stem-group of actinopterygii and sarcopterygii possessed odonotode-like structure in their scales, called ganoine scales and cosmoid scales, respectively. Owing to the stem-group teleostei lost the dermal odontodes, almost no catfish species (Siluriformes) possess the dermal odontodes. While Loricarioidei show regains of dermal odontodes in their evolutionary history. Bone staining pictures and HE-stained transverse sections show the formation of dermal bones in the representative Siluroidei (*Ictalurus punctatus*, TL: 5 cm, bottom, *n* = 2) and Loricarioidei (*Ancistrus sp*., TL: 6 cm, top, *n* = 2). Black and gray boxes indicate gain and loss of dermal odontodes, respectively. *CD* cranial denticle, *DBP* dermal bony plate, *SB* skull bone.

Extant chondrichthyans (cartilaginous fish), some non-teleost actinopterygians (ray-finned fishes, e.g. gars and bichirs), and some sarcopterygians (lobe-finned fish, e.g., coelacanth) retain dermal odontodes on the body surface in the forms of placoid scales or ganoid scales (Fig. 1)^9–12^. However, dermal odontodes were lost in the early divergence of teleosts (the largest group in actinopterygians) and sarcopterygians; thus, most of them no longer have these structures. Intriguingly, dermal odontodes independently re-emerged in four living teleost groups: *Xiphioidae* (swordfish), *Denticeps* (denticle herring), *Atherion* (pickleface hardyhead), and *Loricarioidae* (armored catfish) (Fig. 1)^13–16^. In these fishes, dermal odontodes on the extraoral skin display the conserved odonotode organization and components, e.g., a pulp cavity surrounded by a dentin cone but without an enamel cap. The exceptional resemblance of dermal odontode architectures among these fishes implies redeployment of the genetic network for odontode formation from existing teeth or resurrection of the genetic program for lost dermal odontodes in each lineage, yet little is known about the genetic basis underlying odontode convergent evolution in teleosts.

The developmental process of odontodes and its core set of regulatory genes were uncovered principally by studying mammalian tooth formation. Tooth development begins with epithelial placode formation; the thickened epithelium with reduced cell proliferation functions as a signaling center, expressing the transcription factor *paired like homeodomain 2* (*pitx2*) as well as genes encoding secreted factors, e.g., *sonic hedgehog* (*shh*), *fibroblast growth factors* (*fgfs*), *bone morphogenic proteins* (*bmps*), and *wingless-type MMTV integration site family* (*wnts*)^17–20^. Via these diffusible molecules, the placode triggers the sequential and reciprocal epithelial-mesenchymal interactions and regulates formation of the tooth primordium, i.e., tooth germ, through the well-defined developmental stages of bud, cap, bell, and eruption^21,22^.

Recent comparative studies established histological and genetic links between teeth and various dermal odontodes. For example, placoid scales, the dermal odontodes of cartilaginous fish, form through the strictly conserved bud, cap, bell, and eruption stages^23^. Also, *shh*, *wnt*, *bmp*, *fgf* and other genes indispensable for tooth development are expressed in a comparable manner during both tooth and placoid scale formation^23–26^. These architectural and molecular correspondences between tooth and dermal odontode development supports the classical hypothesis that genetic cascades were co-opted from dermal odontodes to teeth (or vice versa). However, due to the lack of experimental model animals amenable to genetic manipulation, there is little direct experimental evidence for this hypothesis and, more broadly, for how odontodes are recruited into novel tissues.

The catfish order (Siluriformes) offers a remarkable and unique opportunity to understand the evolutionary mechanisms of dermal odontodes diversification. The last common ancestor of catfish is thought to have lost all body scales, but some lineages in this group “Loricarioidea” reacquired dermal odontodes, i.e., dermal denticles, with/without underlying dermal bony plates^15,27^. These dermal denticles in catfishes display the rigorously conserved odontode characteristics albeit lacking surface enamel. The distribution of dermal denticles along the body in these catfish lineages is evolutionarily diverse, occurring on the head, trunk, tail, and peduncle^28^. These distinct lineages with their morphologically-diverse gains of dermal denticles are prominent model systems for studying the genetic mechanisms of dermal odontode evolution. Furthermore, the genomic sequences of three catfish species (*Ictalurus punctatus, Pterygoplichthys pardalis,* *Platydoras armatulus*) with/without dermal denticles were recently assembled, establishing the catfish order as a genetically approachable clade^29^. This combination of unique, yet anatomically conserved, odontode development and available genomic resources make the catfish order ideal for identifying the genetic mechanisms of dermal odontode evolution.

In this study, we established the suckermouth armored catfish (*Ancistrus sp.*; Loricariidae) as an experimental model animal to investigate the evolutionary and developmental mechanisms of dermal odontodes. Our histological studies revealed that cranial dermal denticles continuously arise and develop from embryonic stages to, at least, the early adult stage via the previously defined developmental stages of odontodes. We then compared gene expression profiles of cranial, scute, and oral tissues by de novo transcriptomic assembly and identified evolutionarily conserved elements of a genetic regulatory network for dermal denticle formation. Finally, we revealed that *paired like homeodomain 2* (*pitx2*) is expressed in morphologically characterized dermal denticle placodes as seen in early teeth development of other vertebrates^21^. These results suggest that dermal denticles might have evolved via redeployment of *pitx2* in *Ancistrus sp.* and shed light on how dermal denticles have repeatedly evolved along the vertebrate body.

## Results

### Cranial dermal denticle development in *Ancistrus sp*

**Figure 2:**
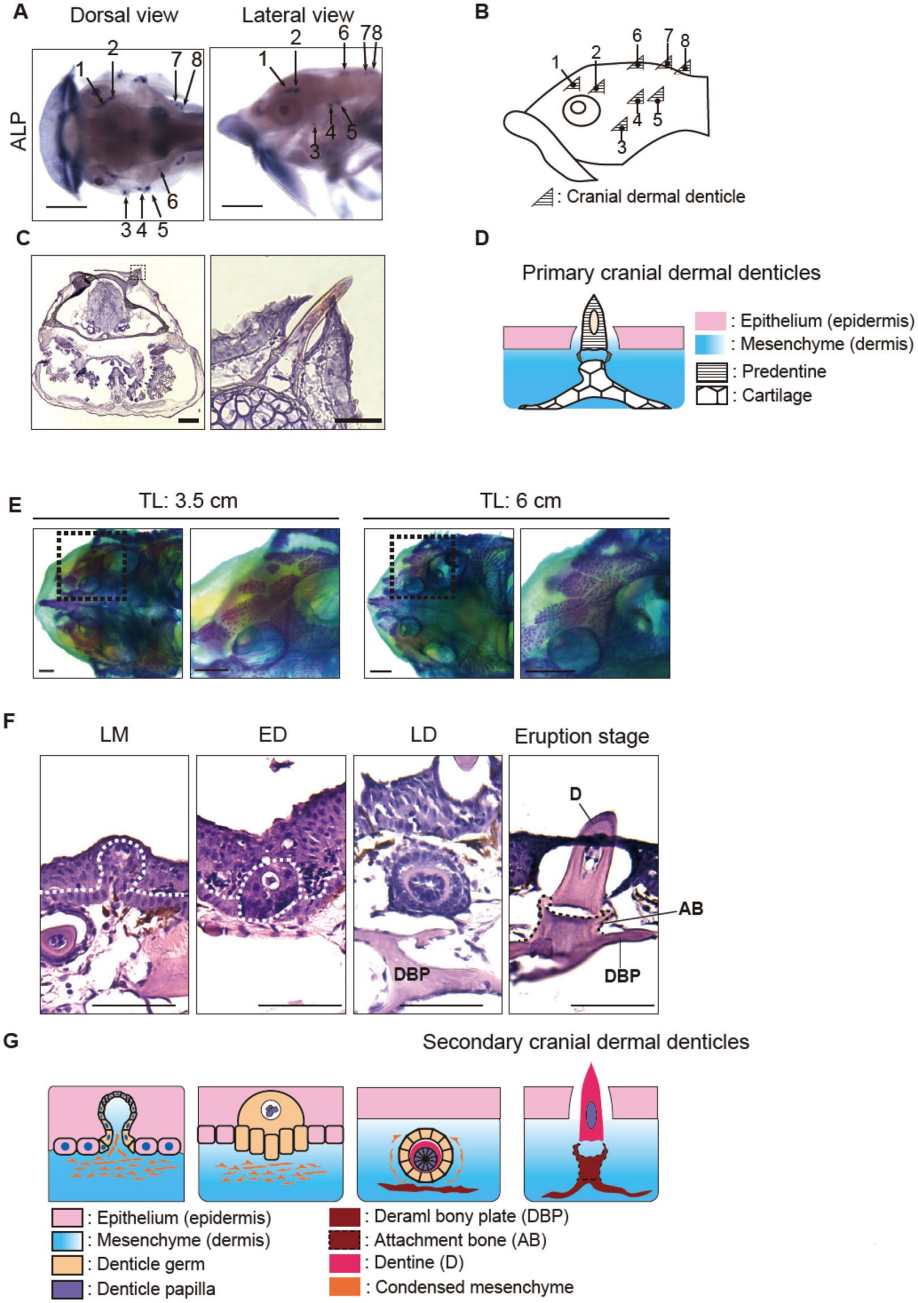
Cranial dermal denticles develop through conserved odontode developmental stages. (**A**) ALP staining shows developing primary cranial dermal denticles. Eight cranial dermal denticles form on both the left and right sides. Scale bars: 0.5 mm. (**B**) Schematic illustration of the position of primary cranial dermal denticles. (**C**) HE-stained transverse section of *Ancistrus sp.* embryo at 8 dpf. Primary cranial dermal denticles form in the cranial epithelium and mesenchyme, being anchored to underling cartilages via the attachment fiber-like structures. Scale bars: 0.1 mm. The right panel shows higher magnification of the dotted box area in the left panel. Scale bars: 0.5 mm. (**D**) Schematic illustration of primary cranial dermal denticle positions (left) and their structure (right). (**E**) Bone and cartilage staining of a juvenile (TL: 3.5 cm) and adult fish (TL: 6 cm). The number of cranial dermal denticles increased with underlying dermal plate expansion from the juvenile to the adult stage. Each right panel shows higher magnification of the dotted box area in the left panels. Scale bars: 1 mm. (**F**) Secondary cranial dermal denticle development through the bud, cap, bell, and eruption stages in adult fish (TL: 6 cm). White dotted lines indicate the boundary between the surface epithelium and cranial dermal denticle germs. Black dotted line indicates the attachment bone. Scale bars: 100 µm. (**G**) Schematic illustration of the developmental stages of secondary cranial dermal denticles. At least two biological replicates were investigated in each experiment. *LM* late morphogenesis, *ED* early differentiation, *LD* late differentiation.

Suckermouth armored catfish are unique among catfish in that they have evolved dermal denticles over most of their bodies except for the abdomen^30^ (Fig. 1, Supplementary Fig. S1). Previous studies showed that dermal denticles in suckermouth armored catfish possess conserved odontode architecture^28^. To examine the process of dermal denticle development, we performed mineralization staining and histological analysis of developing dermal denticles in *Ancistrus sp.*, commonly called the bristlenose pleco and known for its relatively small size and ease of breeding. Cranial dermal denticles were the first denticles to appear and formed on the cranial skin at 96 h post fertilization (hpf) (Fig. 2A) as revealed by alkaline phosphatase (ALP) staining, a marker of odontoblast differentiation^31^. We observed eight symmetrically arranged dermal denticles on both the left and right sides of the cranium (Fig. 2A). These cranial denticles were not stained by alizarin red, reflecting their pre-mineralized state (Supplementary Fig. S2A). At this stage, based on hematoxylin and eosin (HE) staining, the cranial dermal denticles are anchored to underlying cartilages via attachment-fiber like structures, as previously described for the placoid scales of cartilaginous fish (Fig. 2B)^2^ and were shed approximately by 10–14 days post fertilization (dpf) (the early larva stage), thus we term them the primary cranial dermal denticles (Fig. 2C). After 14 dpf when the larvae have consumed almost all yolk contents and begun feeding, secondary cranial dermal denticles appear at new positions on the cranial skin surface (Supplementary Fig. S2B). These denticles as well as the underlying skull roof displayed mineralization based on Alcian blue and Alizarin red staining (Fig. 2D). As the juveniles grew, the number of mineralized secondary cranial dermal denticles increased along with the expansion of underlying dermal bony plates to cooperatively construct the head armor—the most distinctive feature of armored catfish (Fig. 2D, Supplementary Figs. S1, S2B, C). Also, the secondary cranial dermal denticles continuously appeared and matured from ~ 14 dpf (the larval stage) to, at least, 360–540 dpf (the early adult stage, 6–7 cm total length (TL)). In summary, *Ancistrus sp*. develops deciduous primary cranial dermal denticles and consecutive secondary cranial dermal denticles at different developmental time points, and only the latter mineralize and form the head armor.

### The developmental stages of cranial dermal denticles in suckermouth armored catfish

To characterize the developmental stages of the primary and secondary cranial denticles, we examined the histology of dermal denticles at 60 hpf (primary cranial dermal denticles at the embryonic stage), 120 dpf (secondary cranial dermal denticles at the juvenile stage), and 360 – 540 dpf (secondary cranial dermal denticles at the early adult stage). In adult fish, secondary cranial dermal denticles at the late morphogenesis (LM)^23,26^ stage exhibit a thickened basal layer of the epithelium just beginning to invaginate towards the surface epithelium (Fig. 2E). Subsequently, at the early differentiation stage (ED), the epithelium becomes more folded and encloses the denticle papilla, forming a dermal denticle primordia termed the denticle germs^32^. The epithelial cells then completely encircle the denticle papilla where predentin differentiation initiates (the late differentiation stage, LD) (Fig. 2E). The underlying dermal bony plates simultaneously develop in the mesenchymal layer under the denticle germs. Finally, dentin cones erupt from the epithelium, being directly associated with underlying dermal bony plates called attachment bone (the eruption stage) (Fig. 2E)^15^. It was difficult to reliably identify all four developmental stages in embryos and juveniles, but the cap stage was detected in embryos and the cap, bell, and eruption stages in juveniles (Supplementary Fig. S2D). From our histological analysis, we conclude that, in *Ancistrus sp.,* both primary and secondary cranial denticles develop through the conserved odontode developmental stages as previously defined in tooth and placoid scale morphogenesis (Fig. 2E)^6,21,33^.

### De novo transcriptomic profiling of teeth and cranial and trunk denticles in *Ancistrus sp*

Because the developmental stages of *Ancistrus sp.* cranial dermal denticles are shared with those of teeth and placoid scales of other vertebrates, we decided to test whether the denticles also express the key genes of the odontode gene regulatory network (oGRN) in these fish^7^. To comprehensively identify the genes that function in *Ancistrus sp*. odontode development, we conducted comparative de novo RNA sequencing assembly (de novo RNA-seq) of cranial dermal denticles (juveniles and adult), trunk dermal denticles on scutes (adult), and teeth (adult). Because the odontodes are small for manual dissection, we recovered total RNA from cranial skin containing dermal denticles at 120 dpf (juveniles, approximately TL 2–3 cm, “CDj”) and at ~ 360 dpf (sexually matured adult, approximately TL 5–6 cm, “CD”) as well as adult scutes containing trunk dermal denticles (“Scute”), and adult oral epithelial tissues with teeth (“Mouth”) (Supplementary Fig. S3A).

After de novo assembly of short reads from high-throughput sequencing, the contigs were further processed to longer fragments, i.e., unigenes, which were functionally annotated by the NCBI Nr database. First, we investigated the species distribution of homologues for the annotated unigenes and found that 39.8% of the unigenes were homologues of channel catfish genes (*Ictalurus punctatus*) and 17.3% were homologues of red piranha genes (*Pygocentrus nattereri*) (Supplementary Fig. S3B), suggesting that the genes obtained from *Ancistrus sp. *de novo RNA-seq are more similar to those of the catfish family than to those of any other fish species. All assembled unigenes from the developing dermal odontodes of *Ancistrus sp.* were categorized into three Gene ontology (GO) groups: biological process (BP), cellular component (CC), and molecular function (MF) (Supplementary Fig. S3C). In the BP category, 7739 unigenes were assigned to “developmental process” (Supplementary Fig. S3C), confirming that de novo RNA-seq successfully enriched for genes involved in the developmental processes of body structures.

**Figure 3:**
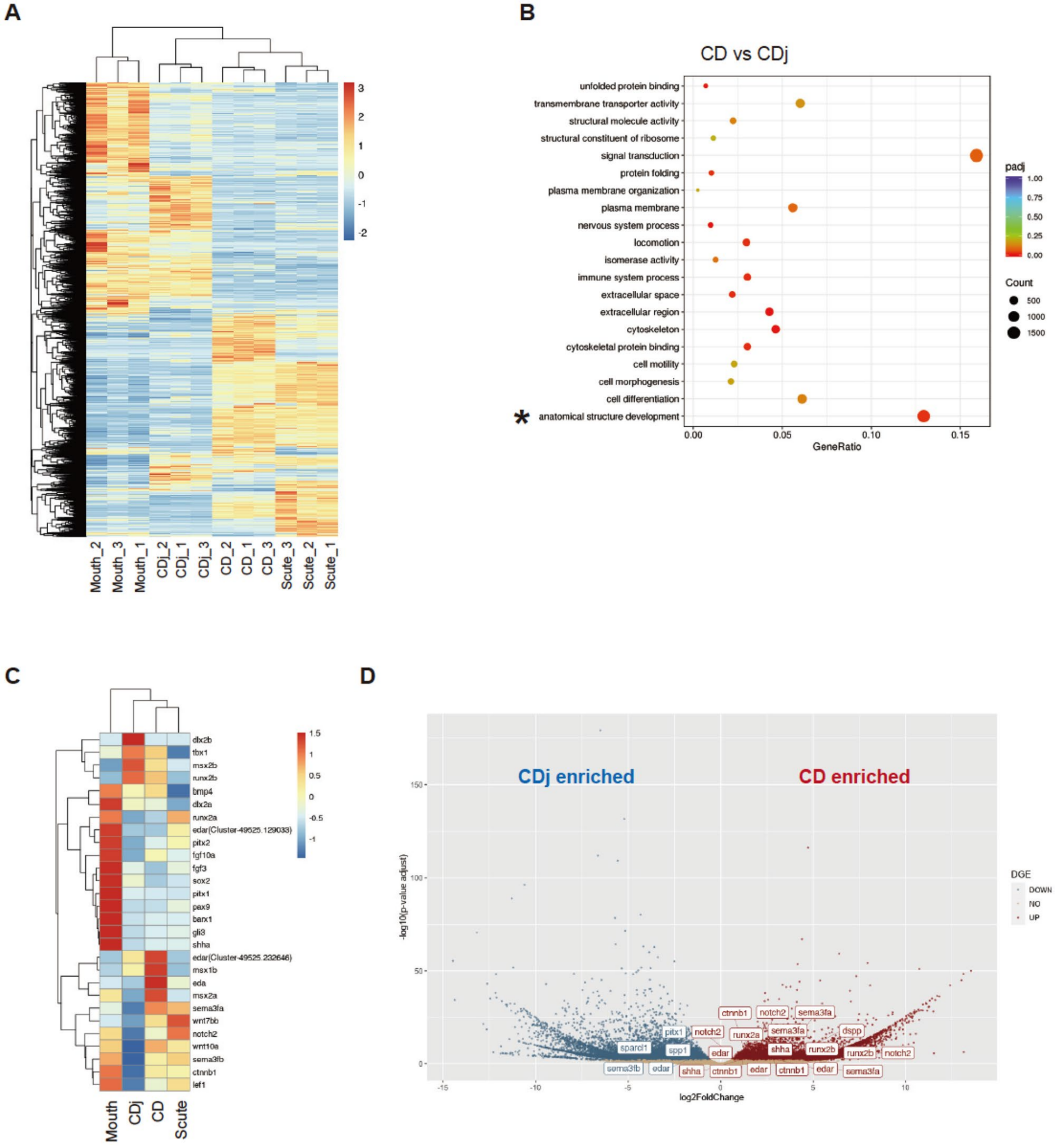
Comprehensive identification of the genes involved in dermal denticle formation of *Ancistrus sp.* Differential gene expression analysis was performed using de novo RNA-seq data from juvenile cranial dermal denticles (CDj, *n* = 3), adult cranial dermal denticles (CD, *n* = 3), Teeth (Mouth, *n* = 3), and scutes (Scute, *n* = 3). (**A**) The heatmap of hierarchical clustering of top 25,000 differentially expressed unigenes. Note that all dermal odontode samples (CDj, CD, and Scute) were clustered together with Mouth as an outgroup. (**B**) GO enrichment analysis of the upregulated genes in CD compared to CDj. The vertical axis is the GO terms, and the horizontal axis is the enrichment of each term. The size of each point represents the number of upregulated genes, and the color of the points represents the adjusted *p*-value. (**C**) The heatmap produced by hierarchical clustering of the expression levels of 27 selected genes involved in oGRN. The color bars indicate the relative expression levels in FPKM. (D) The volcano plot displays the pattern of gene expression values for CD relative to CDj. Significantly enriched genes in CDj are highlighted in blue (log2foldchange < -0.6 and adjusted *p*-value < 0.01) and enriched genes in CD are in red (log2foldchange > 0.6 and adjusted *p*-value < 0.01). The gene symbols show differentially expressed genes associated with oGRN and odontogenesis. As different unigenes were annotated by the same gene names or even the homologous genes in different species, the multiple same gene names were shown in the plot.

Hierarchical clustering of the top 25,000 differentially expressed unigenes across all samples indicated that the gene expression profile of cranial dermal denticles (CDj and CD) is more similar to that of trunk dermal denticles (Scute) than to that of teeth (Mouth) (Fig. 3A). To explore the similarity of functional gene categories among the CD, CDj, Scute, and Mouth samples, we then carried out GO enrichment analysis, comparing CDj to each adult tissue. The analysis showed that GO:0,048,856 “the anatomical structure development”, which includes the gene set associated with odontogenesis, was enriched in all adult odontode tissue (Fig. 3B, Supplementary Fig. S3D, asterisks).

Previous studies indicated that the oGRN is functional in both tooth and dermal denticle development^7^. To test whether the oGRN underlies odontode formation in *Ancistrus sp.*, we examined the expression level of 27 genes previously reported as the central components of the oGRN in early dermal odontode and tooth formation (Fig. 3C). Hierarchical clustering of all samples with only the representative oGRN genes grouped the CDj, CD, and Scute samples together with Mouth as an outgroup (Fig. 3A,C). As we anticipated, oGRN genes were highly enriched in CDj and CD samples, including *msx1b, msx2a/b, dlx2a/b, bmp4,* and *edar.* Notably, *wnt7bb, wnt10a, ctnnb1* (*b-catenin*)*,* and *lef1*, the components of the Wnt/*b* -catenin signaling pathway indispensable for odontode mineralization, were significantly enriched in adult odontodes (CD, Scute, and Mouth samples) (Fig. 3C)^17^. Intriguingly, some samples had distinct expression levels of certain oGRN genes, i.e., *bmp4* and *dlx2a* were highly enriched in CDj, CD, and Mouth but not in Scute. Also, the Mouth sample highly expressed the early odonotogenesis regulators (e.g., s*hha* and *pitx2)* and the dental epithelial stem cell marker (*sox*2), a gene indicative of polyphyodonty (teeth replacement throughout life)^34^. Thus, in suckermouth armored catfish, the conserved oGRN seems to function in early cranial and trunk dermal denticle development as it does during tooth and dermal odontode development in other vertebrates.

Next, to investigate the similarity of the maturation process of teeth and dermal odontodes, we created volcano plots and compared gene expression levels in the CDj, CD, Scute, and Mouth samples (Fig. 3D, Supplementary Fig. S3E). The expression of *sparc-like 1* (*sparcl1*) and *secreted phosphoprotein 1* (*spp1*), which are indispensable for mineralization of dentin and bones^35–37^, were higher in CDj than in CD, Scute, and Mouth (Fig. 3D, Supplementary Fig. S3E). In contrast, *runx2a* (*runt-related transcription factor2a*), *b-catenin* (*ctnnb1*), *edar*, and *notch2*, factors indispensable for odontoblast differentiation^17,38–41^, were enriched in CD, Scute, and Mouth. Moreover, *dentin sialophosphoprotein* (*dspp*), which is abundant in the dentin extracellular matrix^37,42^, was also more highly expressed in these adult samples than in CDj. The function of these genes in dentin mineralization and maturation require more comprehensive investigation. Still, our data implies that Sparcl1 and Spp1 contribute to the early mineralization process of dentin, and DSPP, previously reported to be regulated by the Wnt-Runx2 pathway^39,43^, controls the late phase of odontoblast differentiation and mineralization. Overall, there is conservation between the genetic programs involved in the maturation and mineralization of dermal denticles in suckermouth armored catfish and those programs regulating tooth and dermal odontode development in other vertebrates^35,44^.

### Developing cranial dermal denticles of *Ancistrus sp*. showed evolutionarily conserved expression patterns of oGRN and dentinogenesis genes

**Figure 4:**
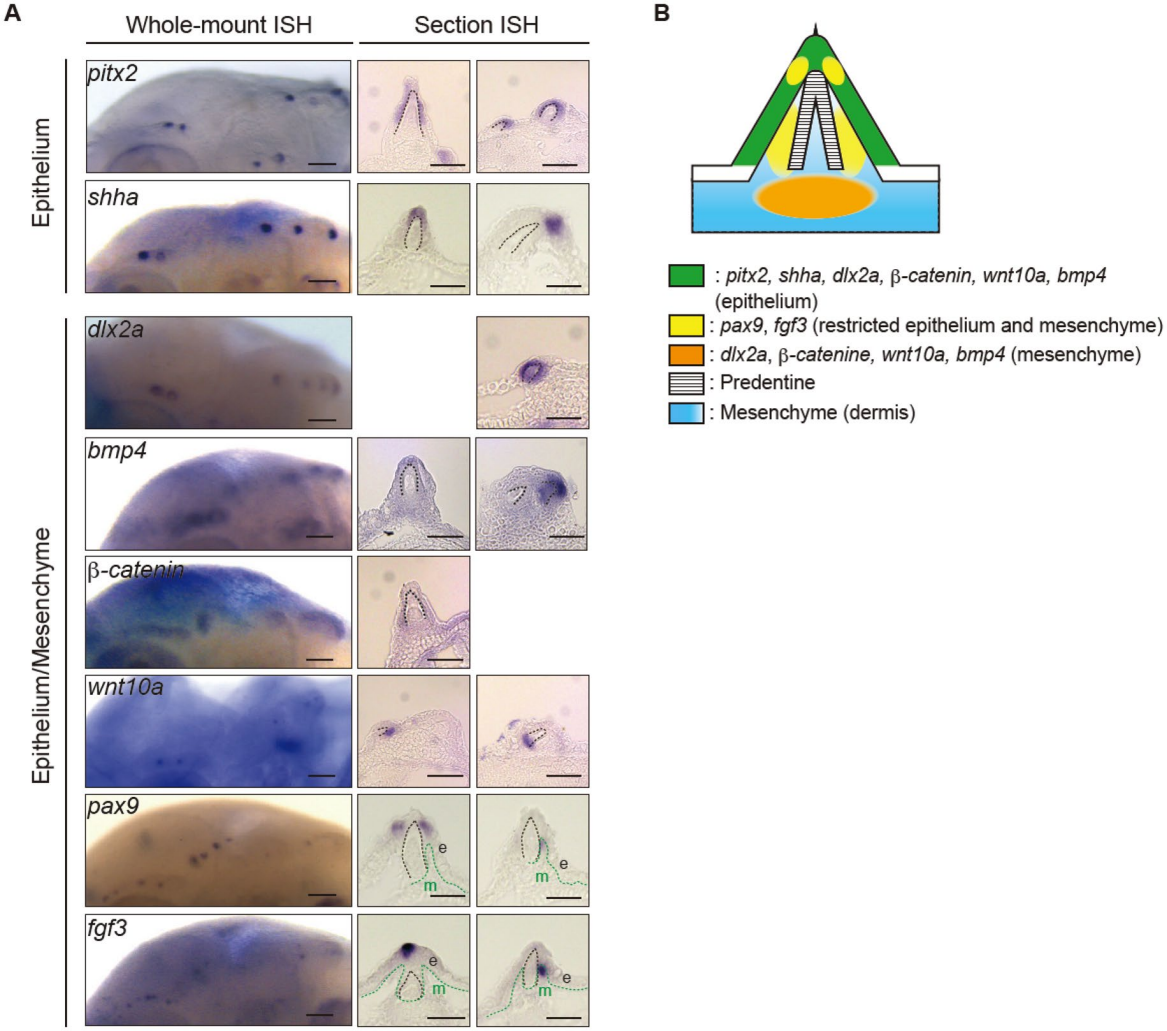
oGRN gene expression in primary cranial dermal denticles. (**A**) In situ hybridization (ISH) for oGRN genes in primarily cranial dermal denticles of *Ancistrus sp.* at 96 hpf. Whole-mount (left) and section (right) in situ hybridization of represented oGRN genes at the eruption stage. *Pitx2* and *shha* transcripts were detected in the epithelium. *Dlx2a, b-catenin, wnt10a, bmp4, pax9,* and *fgf3* transcripts were in both the epithelium and mesenchyme. Black dotted lines indicate the borderline of the epithelium and dentin cone. Green dotted lines indicate the borderline of the epithelial or dentin cone and mesenchyme. For the epithelium genes, the left and right section panels show the entire epithelial and distal tip expression, respectively. In *dlx2a, bmp4, and b-catenin* staining, the section panels show both epithelial and mesenchymal expression (left) and epithelial distal tip expression (right). In *wnt10a, pax9, and fgf3* staining, the section panels show the epithelial tip expression (left) and local mesenchymal expression adjacent to the dentin (right). Note that, to display the representative oGRN gene expression patterns, we selected different slice positions of dermal denticles for some of the section ISH photos. Accordingly, some denticle germ morphology appear to be different from others. Scale bars: 100 µm (left) and 50 µm (right). e; epithelium, m; mesenchyme. (**B**) Schematic illustration of oGRN gene expression patterns in secondary cranial dermal denticles at the eruption stage. At least three biological replicates were investigated in each experiment.

We next performed in situ hybridization (ISH) with RNA probes complementary to selected oGRN genes (*pitx2, bmp4, dlx2a, fgf3, shha, wnt10a, b-catenin,* and *pax9*)^7^. All tested genes were expressed in developing cranial dermal denticles at 96 hpf (primary denticles at the embryonic stage) and at 120 dpf (secondary denticles at the juvenile stage) (Fig. 4A, Supplementary Figs. S4B, S5) as well as in embryonic teeth and dermal denticles on pectoral fin spines (Supplementary Fig. S4). In primary cranial dermal denticles at the eruption stage, the expression of *pitx2* and *shha* was restricted to the epithelium surrounding dermal germs (Fig. 4A, B). Transcripts for *dlx2a*, *β-catenin*, *wnt10a, bmp4,* and *fgf3* were detected in both epithelial and mesenchymal cells (Fig. 4A,B). *Pax9* is expressed in the mesenchyme of developing teeth and placoid scales^7^, but here was found in both epithelial and mesenchymal cells (Fig. 4A, B). The expression domains of *fgf3* and *pax9* expression domain were shown to be associated with the direction of denticle eruption in shark teeth, and the eruption of cranial dermal denticles may also be regulated by these molecules^45^. These results suggested that, despite minor variants, the oGRN genes controlling dermal odontode development in suckermouth armored catfish exhibit patterns comparable to those previously reported for other vertebrate odontodes^6,7^.

Our RNA-seq data discerned dentinogenesis-related transcripts, such as *dspp*, *spp1*, and *sparcl1*, in juvenile and adult cranial denticle samples (Fig. 3D). We thus validated the spatial distribution of these transcripts using 96 hpf embryos by ISH and revealed that *dspp*, *spp1*, and *sparcl1* were expressed in cranial dermal denticles, teeth, and pectoral fin spines (Supplementary Fig. S6). Taken together, oGRN and dentinogenesis-related gene expression contributes to the odontoblast differentiation and dentin mineralization throughout the developmental stages.

### *Pitx2* is an early marker of dermal denticle development in* Ancistrus sp*

**Figure 5:**
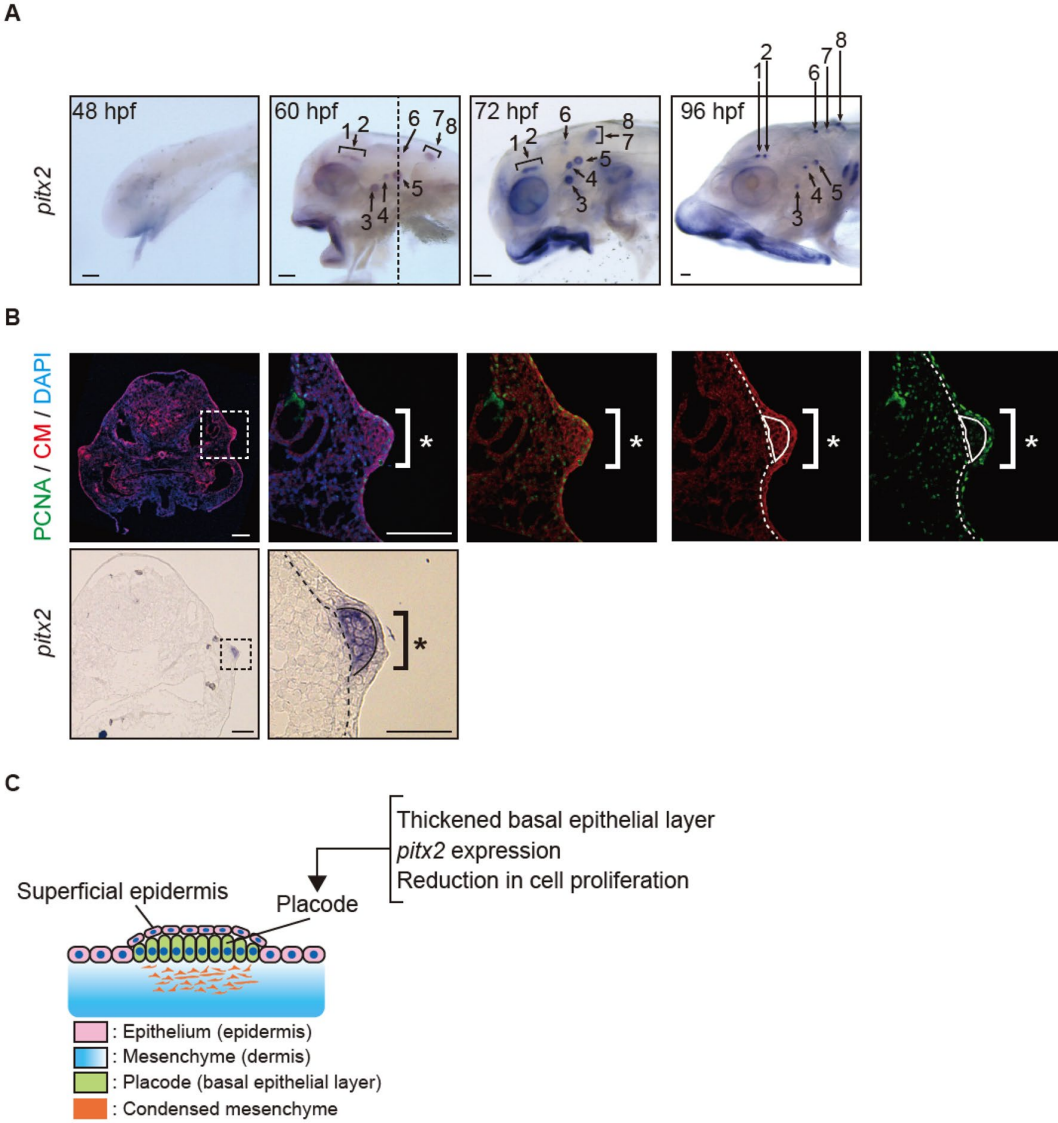
Anatomically and molecularly defined epithelial placode for cranial dermal denticles. (**A**) Whole-mount in situ hybridization of *pitx2* in *Ancistrus sp.* embryos at 48, 60, 72 hpf, and 96 hpf. *Pitx2* expression in developing dermal denticles was discerned at 60 dpf. Each number indicates cranial dermal denticles and the numbering is consistent in all panels. Scale bars: 100 µm. (**B**) Immunostaining of PCNA (green) and cell membrane (DiI labeling, red) with nuclear staining (DAPI) (top), and in situ hybridization of *pitx2* for transverse sections at the position of the dotted line in A (bottom). *Pitx2* transcripts were detected in the anatomically defined placode (white brackets; thickened epithelial tissue with the reduction of cell proliferation). White and black dotted lines indicate a boundary between epithelium and mesenchyme. Black solid line in *pix2* panel indicates a boundary between the top and bottom layer of epidermis. Scale bars: 100 µm. (**C**) Schematic illustration of the cranial dermal denticle placode. At least three biological replicates were investigated in each experiment.

Our de novo RNA-seq and ISH identified *pitx2, dlx2a*, and *bmp4* in the epithelium of dermal germs at the eruption stages in *Ancistrus sp.* (Figs. 3, 4, Supplementary Figs. S4, S5). Expression of these genes in the dental placode contribute to the initiation of tooth germ development by inducing the epithelial thickening^46,47^. Thus, we wondered whether teeth developmental program had been redeployed into the cranial skin in the course of cranial dermal denticle evolution in suckermouth armored catfish. To test this hypothesis, we dissected the induction mechanisms of the epithelial placode for cranial dermal denticles by examining the expression patterns of *pitx2*, *dlx2a*, *bmp4, pax9,* and *shha,* which are early regulators of tooth epithelial placodes^21^. At 60 hpf, among these genes, only *pitx2* expression was detected in the developing primary cranial dermal denticles (Fig. 5A and Supplementary Fig. S7A, B) and by 72 hpf, expression of *dlx2a* and *bmp4* were also present (Supplementary Fig. S7C). From 48 to 72 hpf, we could not detect the denticle expression of other oGRN genes. e.g., *pax9* or *shha,* in denticles. These genes are reported to be expressed slightly later than *pitx2* in mammalian tooth development (Supplementary Fig. S7C)^48^. We observed a similar stepwise expression pattern of *pitx2, dlx2a,* and *bmp4* in developing teeth and dermal denticles on pectoral fin spines (Supplementary Fig. S7A–C). Intriguingly, to date, *pitx2* expression has not been detected during dermal odontode development except for teeth^23^.

To further characterize the placode formation during cranial dermal denticle development, we performed immunostaining for proliferating cell nuclear antigen (PCNA). A reduction in this stain marks the initial placode formation^24,49^. In *Ancistrus sp.* embryos, the epithelium is segregated into two layers at 60 hpf as seen in teeth development of other jawed vertebrates^21^; superficial epidermis layer and thickened basal layer, which forms the cranial dermal denticle placode. PCNA immunostaining revealed reduced cell proliferation in the thickened basal layer relative to the superficial epidermis and the dermis that underlies the basal layer (Fig. 5B). Cryosectioning of embryos used for *pitx2* ISH embryos revealed that *pitx2* was also enriched in the thickened basal epithelial layer (Fig. 5B). These results demonstrated that the anatomically defined placodes with the localized *pitx2* expression underlie cranial dermal denticle formation in *Ancistrus sp.* (Fig. 5C).

### Pitx2 plays an essential role in the formation of cranial dermal denticle placodes

Based on our ISH results, *pitx2* is the earliest gene to be expressed in the epithelium of *Ancistrus sp.* dermal denticles. Pitx2 is an inducer of the mammalian tooth epithelial placode^47^, thus, could also establish placode formation by inducing oGRN gene expression and underly the head armor evolution in *Ancistrus sp.* Given that *pitx2* is required for the development of teeth, eyes, pituitary glands, muscles, lung, and heart in vertebrates, whole-body genetic deletion of *pitx2* could result in embryonic lethality before dermal denticles develop^50^. Although mosaic knockout of *pitx2* gene with Cas9 in F0 fish is a great approach to reveal Pitx2 function in cranial dermal denticle formation, a complete elimination of *pitx2* in a certain number of cells in essential organs may lead to embryonic lethality before cranial dermal denticles form. To circumvent this possibility, we targeted the *pitx2* mRNA using CRISPR-RfxCas13d, which was recently reported as a novel gene knockdown tool for targeting maternal and zygotic mRNAs^51^. CRISPR-RfxCas13d system has been shown to be more specific to target mRNA compared with conventional knockdown systems (e.g. shRNA or morpholino)^51,52^. We injected RfxCas13d protein with/without three guide RNAs complementary to different sites of *pitx2* mRNA (gRNA#1–3) into one- or two-cell stage embryos (Supplementary Fig. 8A). By 72 hpf, the mRNA level of *pitx2* and its transcriptional target *dlx2a*^46^ in knockdown embryos was 50% of that of control embryos as confirmed by real-time PCR (Supplementary Fig. 8B). Knockdown embryos exhibited smaller and thinner placodes than control embryos (Supplementary Fig. 8C), and, based on ISH, these thinned placodes showed a marked low detection of *pitx2* transcripts (Supplementary Fig. 8C). At 96 hpf, both the staining intensity of the odontoblast differentiation marker ALP and the number of dermal denticles were decreased in knockdown embryos (Supplementary Fig. 8D). Although all cranial dermal denticles in *pitx2*-KD embryos emerged at the same timing as control embryos, some of them showed severe defects compared to control specimens (Fig. 5A, Table S2). These partial defects in a single embryo suggested that *pitx2*-KD specifically affected the cranial dermal denticle development, not the entire cranial tissues. Finally, to test whether *pitx2* triggers oGRN gene expression, we investigated the transcript levels of *dlx2a*, *bmp4,* and *dspp* and found that *pitx2* knockdown embryos exhibited reduced expression of these genes in cranial dermal denticles, teeth, and denticles on pectoral fin spines (Supplementary Fig. S8E and S9). These results suggest that *pitx2* may induce oGRN gene expression and is responsible for forming the placode of dermal odontodes in suckermouth armored catfish.

## Discussion

**Figure 6:**
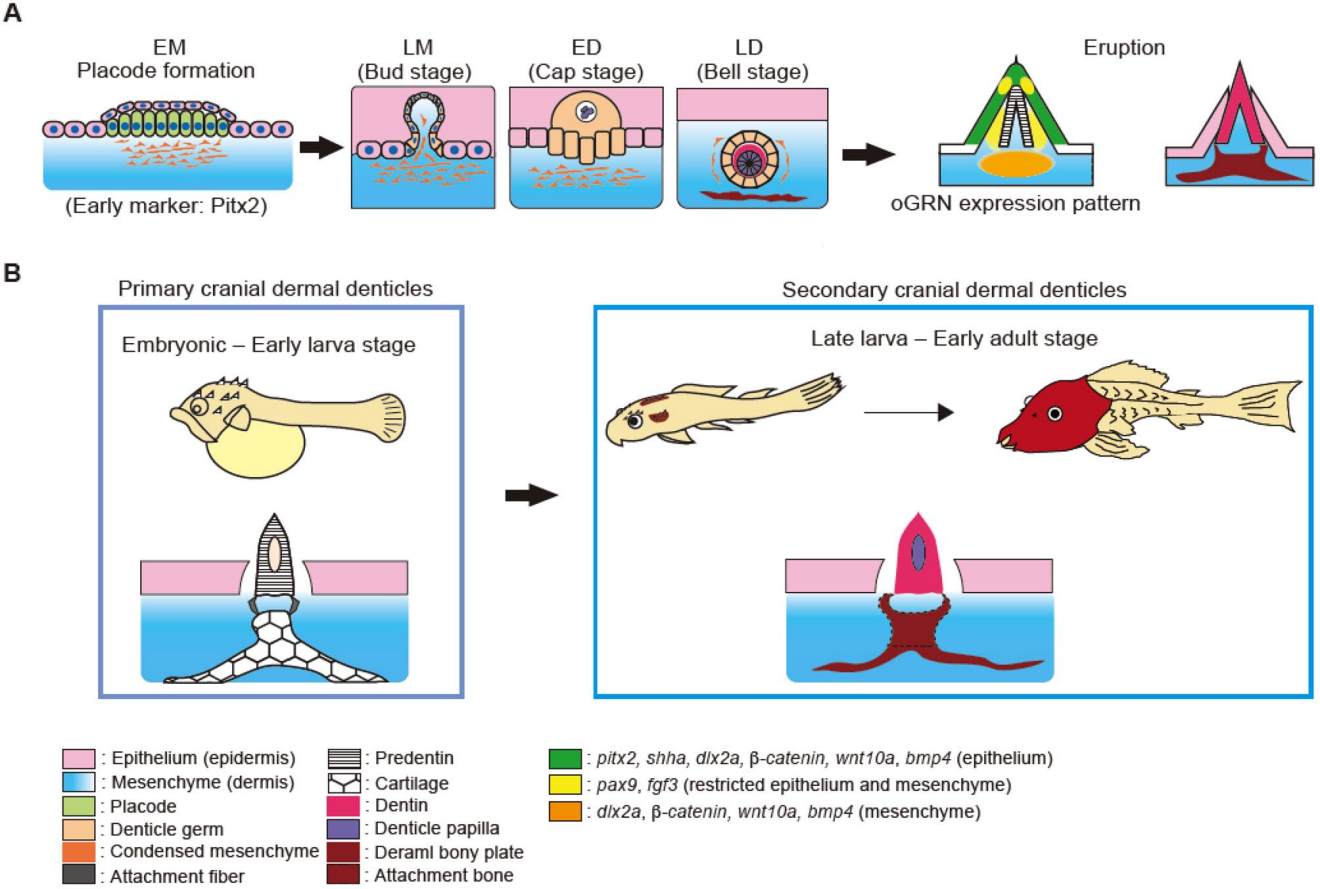
The developmental process of cranial dermal denticles in *Ancistrus sp.* (**A**) Cranial dermal denticle morphogenesis progresses through the evolutionarily conserved odontode developmental stages. First, the cranial epithelium thickens to form the epithelial placode. The placode invaginates and forms a bud-shaped epithelial layer (*EM* early morphogenesis). Subsequently, condensed mesenchymal cells invade the lumen of the denticle germ, leading to the formation of denticle papilla (*LM* late morphogenesis). The denticle germ forms circular epithelial tissue enclosing the denticle papilla (*ED* early differentiation). The denticle papilla then differentiates into dentin inside the denticle germ, forming a bell-shaped structure (*LD* late differentiation). At this stage, underlying dermal bony plates differentiate underneath the dental germ. LM, ED, and LD may correspond to the bud, cap, and bell stage of tooth development, respectively. Dentin finally erupts from the epithelium, being anchored to dermal bony plates (eruption stage). The development of primary and secondary cranial dermal denticles is regulated by evolutionarily conserved oGRN. Among oGRN, *pitx2* exhibits the earliest expression in the placode and induces the development of cranial dermal denticles. *Pitx2* and *shha* are expressed in the epithelial layer surrounding the dentin cone. Dlx2a, *Wnt10a, b-catenin,* and *bmp4* are broadly expressed in both the epithelium and underlying mesenchyme. *Pax9* and *fgf3* transcripts were detected in the epithelium and mesenchyme laterally to the dentin cone. (**B**) The formation of cranial dermal denticles in the course of *Ansitrus sp.* development. From the embryonic to the early larva stage (14 dpf), primary cranial dermal denticles form in the restricted positions of the cranial epidermis. After primary cranial dermal denticles shed and disappear, secondary cranial dermal denticles begin to form. The number of secondary cranial dermal denticles increases along with the expansion of underlying dermal bony plates, constructing the head armor.

We comprehensively examined the developmental process of cranial dermal denticles in suckermouth armored catfish. In this system, cranial dermal denticles develop through the histological stages of EM (placode formation), LM (bud), ED (cap), LD (bell), and eruption as previously defined in tooth and placoid scale formation (Fig. 6A)^22,23,33^. In particular, the processes of the epithelial folding, dentin differentiation, and dentin eruption closely resemble events previously shown to occur during tooth development (Figs. 2, 6). These findings accord with those of a concurrent investigation of a different species of suckermouth armored catfish *Ancistrus triradiatus* (Loricariidae; Siluriformes)^53^, and the histological similarities of dermal denticle and tooth formation in both these species implies that dermal denticles in suckermouth armored catfish evolved via redeployment of a preexisting developmental program for teeth in the common ancestor of the catfish family.

The above histological observations are further validated by the genetic studies. Our comparative de novo RNA-seq analysis identified a conserved oGRN for odontode development in suckermouth armored catfish (Fig. 3)*,* and we found that the expression patterns of representative oGRN genes are strictly conserved in dermal denticles and other odontodes, e.g., teeth and placoid scales (Fig. 4)^6,7,23^. However, there were some discrepancies. For example, during tooth formation, *pax9* expression is confined to mesenchyme but it is expressed in both the epithelium and mesenchyme of cranial dermal denticles in *Ancistrus sp.*^6^. Therefore, the epithelial expression of *pax9* could be a derived characteristic in suckermouth armored catfish denticle development.

Notably, the genetic programs of the epithelial placode could have diversified from their evolutionarily ancestral form. For instance, during mouse taste bud development, which likely retains the ancestral developmental program of the epithelial placode^7^, *pax9* is expressed in both the epithelium and mesenchyme^54^. Teeth might have evolved by a co-option of sensory structure developmental programs (e.g. taste buds)^7,55^ and *pax9* expression might have been lost from the epithelium during tooth evolution. Thereafter, epithelial *pax9* expression might be regained during evolution of dermal denticles in suckermouth armored catfish. Thus, the oGRN might have been prone to recurrent modifications during odontode evolution, producing morphological diversification. Overall, our de novo RNA-seq and ISH results highlight the evolutionarily shared and derived genetic characteristics of dermal odontode development in suckermouth armored catfish. These results suggest that heterotopy, such as redeployment of oGRN from teeth into the trunk epidermis, and successive modifications of epidermal appendage developmental programs have produced extraordinarily diverse exoskeletal architectures in extinct and living taxa.

Typically, dermal odontodes form anchored to underlying dermal bony plates, excepting the placoid scales of cartilaginous fish which form without underlying bony supports^2^. Historical hypotheses have proposed that, during dermal odontode development, an underlying dermal bony plate fused with adjacent plates and evolved into large osseous plates. This could be the evolutionary origin of exoskeletons, such as scales or scutes^5,56^. However, these hypotheses have never been functionally tested due to the lack of developmentally amenable model organisms. In *Ancistrus sp.*, we found that primary cranial dermal denticles form anchored to underlying cartilages and secondary cranial dermal denticles develop with underlying dermal bony plates (Fig. 2, Supplementary Figs. S1, S2). Intriguingly, the developmental topology of mammalian teeth and the alveolar bone called the tooth-socket is analogous to that of dermal denticles and their underlying support. During tooth development, the dental lamina, which expresses *sema3f.* and *fgf8*, induces a mesenchymal condensation that gives rise to the alveolar bone^57,58^. Dermal denticles could induce dermal bony plate formation in catfish in an analogous manner considering the similarity of gene expression profiles in tooth and denticle germs (Fig. 3C)^48^. Further research is required to understand the developmental relationship between dermal denticles and their underlying dermal bony plates, but dermal denticles could very well be a main driver in regulating dermal bony plate development and divergence of the head armor. However, some Loricarioidea catfishes possess dermal bony plates without dermal denticles^28^; thus, the developmental relationship of dermal denticles and basal bony plates could be a derived characteristic gained in the course of Loricariidae fish evolution.

Our de novo RNA-seq and subsequent ISH also identified genes likely necessary for maturation and mineralization of dermal denticles. The expression levels of *sparcl1* and *spp1* are higher in juvenile than in adult dermal denticles. *Sparcl1* and *spp1* belong to the secretary calcium binding phosphoprotein (SCPP) family, and *spp1* is thought to have originated from the common ancestral gene *sparcl1* by tandem gene duplication^59^. *Sparcl1, spp1,* and other genes in the SCPP family exhibit diverse expression patterns in dentin odontoblasts and bone osteoblasts and promote mineralization in various vertebrates. For instance, in vertebrates, *SPARCL1* and the sparcl1-orthologue *SPARC* (also called osteonectin) are exclusively expressed in odontoblasts^29,35,59^. However, while fish *spp1* is highly expressed in both odontoblasts and osteoblasts, mammalian *SPP1* is more highly expressed in immature osteoblasts than in odontoblasts^29,35,59^. It remains unclear how *sparcl1* and *spp1* function during odontogenesis but they likely contribute to the early mineralization of cranial dermal denticles in suckermouth armored catfish.

In contrast to *sparcl1* and *spp1, b-catenin* and *runx2* are more highly expressed in adult odontodes samples (adult dermal denticles, teeth, and scutes) than in juvenile samples (Fig. 3C,D, Supplementary Fig. S3E). The Wnt/b-catenin signaling pathway plays a central role in mouse tooth morphogenesis. In that system, Wnt signaling regulates odontoblast development by stimulating transcription of *runx2*, a gene essential for inducing odontoblast differentiation^39–41^, via direct binding of *b-catenin* to the *runx2* promoter together with the Tcf1 transcription factor and induces its expression^39^. Runx2, in turn, binds to the promoter region of *dspp*, another SCPP family member and indispensable for mineralization, and upregulates its transcription^44^. Our RNA-seq data indicates that *dspp* transcripts are enriched in adult cranial dermal denticles (Fig. 3D, Supplementary Fig. S3E) along with *b-catenin* and *runx*2. Thus, a cascade identical or similar to the Wnt/b-catenin–Runx2–DSPP pathway identified in mice likely promotes odontoblast maturation in cranial dermal denticles of suckermouth armored catfish. Intriguingly, we detected *sparcl1* and *spp1* but not *dspp* transcripts in pectoral fin spines at embryonic stages (Supplementary Fig. 6). This implies the relatively late maturation of dermal denticles in pectoral fin spines, compared to teeth and cranial dermal denticles. Overall, our comparative transcriptomic analysis is the first systematic and comprehensive identification of genes involved in the early and late maturation process of dermal odontodes.

*Pitx2* expressed in the epithelial placode initiates tooth germ formation in mammals, but *pitx2* expression has not been detected in developing dermal denticles of cartilaginous fish^23^. Consistent with the concurrent investigation of the oGRN in another species of suckermouth armored catfish^53^, our comparative de novo RNA-seq and histological analysis of *Ancistrus sp*. found that *pitx2* was the earliest oGRN gene to be expressed in developing cranial and trunk dermal denticles (Figs. 4, 5, Supplementary Fig. S7D). Moreover, the knockdown of *pitx2* in suckermouth armored catfish resulted in the cranial dermal denticle defects with decreased expression of *dlx2a* and *bmp4* (Supplementary Figs. S8 and S9). These results are consistent with prior studies which showed that reciprocal interactions between Pitx2, Dlx2, and Bmp4 are required to initiate mammalian tooth germ formation^46,47^. Intriguingly, our molecular phylogenetic analysis indicated that shark Pitx1 sequence is relatively close to Pitx2 compared with that of other vertebrate species (Supplementary Fig. S10). Given that *pitx1* is expressed in developing teeth and scales, Pitx1 may compensate the function of Pitx2 in dermal denticle development in cartilaginous fish^23^. Overall, we may propose that Pitx2 initiates induction of the epithelial placode by triggering oGRN gene expression, including *dlx2a* and *bmp4*, in suckermouth armored catfish (Supplementary Fig. S8).

A recent phylogenetic study suggested that Loricarioidea*,* a catfish suborder including Loricariidae (suckermouth armored catfish) and Callichthyidae (*Corydoras*), independently re-emerged dermal denticles on head and trunk surfaces^28^. However, the underlying genetic mechanisms of this evolution remain elusive. Based on the early expression of *pitx2* and its ability to induce placodes in dermal denticles, we hypothesize that *pitx2* expression might have been redeployed from teeth to dermal denticles in suckermouth armored catfish. Coincidently, the cis-regulatory elements of *pitx1*, another member of *paired-like homeodomain* family, involved in the convergent evolution of fish pelvic fins are under active investigation^60,61^. It was found that recurrent mutations within the fragile *Pel* element, a specific enhancer of *pitx1,* underlie convergent loss of pelvic fins in sticklebacks^62^. It is possible that specific regulatory elements of *pitx2* are more susceptible to mutation than regulatory elements of other oGRN genes and this susceptibility contributed to the redeployment of the tooth developmental program into dermal denticles in catfish*.* Further analysis of dermal denticle evolution in suckermouth armored catfish will help reveal the genetic basis of convergent evolution of dermal odontodes and, possibly, provide us clues to decipher a broader enigma—the evolutionary origins of teeth and dermal odontodes in vertebrate ancestry.

## Materials and methods

### Fish husbandry

Channel catfish (*Ictalurus punctatus*) were commercially purchased from a local fish farm (Live Aquaponics, Florida, USA). Suckermouth armored catfish (*Ancistrus sp.*) were commercially purchased from a local aquarium store and were bred in aquarium tanks. As suckermouth armored catfish has been interbred in commercial aquarium stores, an official identification of species name is not possible. Water was replaced twice a week with dechlorinated water prepared from tap water via chlorine remover treatment (API, tap water conditioner). The water was maintained at 25 °C and pH 6.5. After 14 dpf, juveniles and adult fish were fed a boiled vegetables and algae wafer (Tetra) diet. Each tank contained one male and up to two female fish allowing natural breeding. To promote mating, fish caves, which help male fish maintain territories, were installed into the tanks. Males showed typical mating behavior; they repeatedly invited female fish to enter the caves for 3 to 6 h. After spawning, eggs were transferred into quarantined mesh cages installed in the main tanks, and fries were reared there until 60 dpf. For the following experiments, embryos, juveniles, and adult fish were euthanized with 0.016% MS-222 and fixed as indicated. All animal experiments comply with all relevant ethical regulations and guideline for animal study, were approved by the animal committees of Rutgers University (protocol no. 201702646) and Institutional Animal Care and Use Committee (IACUC). All animal experiments were performed in accordance with ARRIVE guidelines (https://arriveguidelines.org/).

### De novo RNA sequencing

Mouth, cranial skin, and scute samples were manually dissected from *Ancistrus sp.* juvenile and adult specimens, frozen in liquid nitrogen, and ground into a fine powder by a mortar and pestle. Total RNA was isolated from each powdered sample using the RNase Plus Universal Kit (Qiagen) according to the manufacturer’s instruction. RNA quality assessment, library construction, high-throughput sequencing, and de novo transcriptome assembly were conducted by Novogene Co., Ltd (Tianjin, China). Briefly, RNA purity and integrity were measured by an Agilent 2100 Bioanalyzer. Sequencing libraries were constructed with a TruSeq stranded mRNA sample preparation Kit (Illumina), and high-throughput sequencing was carried out to generate paired-end 150 bp reads using the NovaSeq 6000 Reagent Kit (illumina). A total of 1,254,277,785 raw reads were obtained from twelve libraries derived from three replicates each of juvenile cranial dermal denticle (CDj), adult cranial dermal denticle (CD), adult scutes (Scute), and adult teeth (Mouth) samples. The raw reads were filtered to remove adapters, reads containing more than 10% equivocal bases, and reads with base quality less than 20 (Quality score ≤ 20). The clean reads were then de novo assembled by Trinity software and 717,406,047 unigenes were identified. Of these unigenes, 488,726 were functionally annotated by aligning them to the Nr (NCBI non-redundant protein sequences, E-value < 1e-5), Nt (NCBI nucleotide sequences, E-value < 1e-5), Pfam (E-value < 0.01), KOG/COG (Eukaryotic Orthologous Groups/Cluster of Orthologous Groups of proteins, E-value < 1e-5), Swiss-Prot (E-value < 1e-5), KEGG (Kyoto Encyclopedia of Genes and Genome, E-value < 1e-5), and GO (Gene Ontology, E-value < 1e-6) databases. Gene functional classification was performed with BLAST2GO based on the GO annotations. Coding sequences (CDS) for unigenes were predicted with BLAST and ESTScasn (3.0.3). To calculate the expression level, the clean reads were mapped against the de novo assembled transcriptome using Bowtie. The mapping results of Bowtie were analyzed by RSEM, and the read count and fragments per kilobase of transcript sequence per million mapped reads (FPKM) value were obtained for each sample. The count data was then used as the input for Bioconductor package (3.12) edgeR and DEseq2 to test for differential gene expression. GO enrichment analysis of differentially expressed genes (DEGs) was performed using topGO. Raw reads data is available in the NCBI Sequence Read Archive (SRA) database: PRJNA731927.

### Histological analysis

Bones and cartilage in *Ancistrus sp.* specimens were stained by Alcian blue 8GX (Sigma) and Alizarin red S (Sigma) as previously described^63^. Alkaline phosphatase (ALP) staining was performed with BM-purple (Roche) as previously described^64^. Paraffin sectioning was performed by the Research Pathology Services of Rutgers University. Briefly, juveniles and adult fish were fixed by 4% PFA at 4° C for one to two weeks and then decalcified in 10% EDTA at 4° C for one to two weeks. After decalcification, samples were dehydrated through a graded ethanol series, cleared in xylene, and embedded in paraffin. Serial Sections (8–10 µm) were collected and stained by standard hematoxylin and eosin (HE) staining. Bone-stained samples and HE-stained sections were imaged on an M205 FCA (Leica) stereoscope and Eclipse E800 microscope (Nikon), respectively, equipped with cameras.

### Immunohistochemistry

Fixed samples were washed in PBS, immersed in 15% followed by 30% sucrose in PBS at 4° C, and frozen in OCT embedding compound (Sakura). Frozen Sections (8–10 µm) were prepared and treated with an antigen retrieval solution (sodium citrate buffer;10 mM sodium citrate, pH6.0) as previously described^65^. Sections were then permeabilized in 0.3% Triton X-100 in PBS for 15 min and blocked in blocking buffer (5% sheep serum and 0.1% Tween-20 in PBS) for 1 h. The PCNA (CST) antibody was diluted in blocking buffer (1:500) and incubated with the sections overnight. Then, sections were washed in 0.1% Tween-20 in PBS and incubated with a secondary antibody (Alexa Fluor 488, Molecular Probes) and DiI (Vybrant DiI Cell-Labeling Solution, Invitrogen) in blocking buffer (1:1000) for 1 h at room temperature (RT). Counter nuclear staining was performed with DAPI in blocking buffer (1:2000). Images were captured with the LSM 510 Meta inverted confocal microscope (Zeiss).

### In situ hybridization

Based on the CDS prediction for the de novo transcriptome, cloning primers (Table S1) were designed to amplify RNA probes from cDNA. Probes were inserted into the pCR-Blunt II-TOPO vector or pCRII-TOPO vector (Invitrogen). DIG-labeled RNA probes were synthesized with the Riboprobe Systems (Promega) and DIG RNA Labeling Mix (Roche). Whole mount in situ hybridization (ISH) was performed as described^66^.

Before rehydration, 30 dpf juveniles were treated with 6% hydrogen peroxide in methanol for 30 min at RT. After rehydration, embryos and juveniles were treated with Proteinase-K (10 µg/ml) in PBS for 15 min at RT and 30 min at 37° C, respectively. Samples were fixed with 0.5% glutaraldehyde plus 4% PFA in PBS for 20 min at RT and then the hybridization was performed.

Paraffin and frozen sections were prepared as described above and subjected to ISH as previously described^67^. Paraffin sections were rehydrated (or frozen sections washed in PBS) followed by permeabilization in 0.1% Triton X-100 in PBS for 30 min at RT. Tissue sections were equilibrated in 0.1 M triethanolamine with 0.25% acetic anhydride and were hybridized with an RNA probe at 70° C overnight. The signal was detected with an anti-DIG-AP Fab fragment (Roche) and BM-purple, and images of whole mount ISH or section ISH were captured with an M205 FCA (Leica) stereoscope or Eclipse E800 microscope (Nikon), respectively.

### Designing of guide RNA targeting pitx2 and preparation of gRNA-RfxCas13d solution

CRISPR-RfxCas13d knockdown was performed as described previously^51^. Briefly, the pET-28b-RfxCas13d-His vector (Addgene, Plasmid #141,322) was used for RfxCas13d protein production (Bon Opus Biosciences, LLC). The final concentration of RfxCas13d was adjusted to 3 µg/µl in storage buffer (50 mM HEPES–KOH, pH 7.5, 250 mM KCl, 1 mM DTT, 10% Glycerol) and stored at − 80 °C. For gRNA synthesis, the Pitx2 CDS of *Ancsitrus sp.* was analyzed for high accessibility sites using RNAfold software (http://rna.tbi.univie.ac.at//cgi-bin/RNAWebSuite/RNAfold.cgi) and identified 22 nucleotides to generate each guide RNA (gRNA). A gRNA universal forward primer containing a T7 promoter and reverse primers containing target sites are shown in Table S3. gRNA templates were generated with PCR using a pool of 3 different gRNA primers at equal concentrations and were in vitro transcribed using the mMESSAGE T7 Kit (Invitrogen). Note that gRNAs #1–3 were co-transcribed in the same vial. The final concentration of the mixed gRNA solution was adjusted to 1600 ng/µl.

### Suckermouth catfish microinjection

To generate the ribonucleoproteins (RNPs), 1 µl of the mixed gRNA solution (1600 ng/µl) and 4.5 µl of RfxCas13d protein (3 mg/ml) were mixed in 1 µl of nuclease-free water with phenol red (Invitrogen). One to 5 nL of RNPs were injected into one- to two-cell stage *Ancistrus sp.* embryos according to the zebrafish standard method. Briefly, fertilized eggs were transferred to a petri dish and washed with distilled water. Eggs that stuck together were carefully detached using fine tweezers. After RNP injection, embryos were incubated for 24 h in 0.0001% methylene blue plus distilled water in a 28 °C incubator with aeration and then were transferred into a mesh cage installed in the main tanks.

### qRT-PCR

Total RNA was purified from Ctrl and *pitx2*-KD embryos at 72 hpf using the RNeasy Plus Universal Kit (QIAGEN), and cDNA synthesis was performed using the iScript cDNA Synthesis Kit (Bio-Rad). qRT-PCR was performed with a SYBR Green PCR Master Mix and the 7900 Real time PCR system (Applied Biosystems). Results were analyzed by the standard curve method and normalized to b-actin expression. Three biological replicates were investigated in this experiment. Primer sequences are shown in Table S3.

### Phylogenetic analysis

Representative coding sequences (CDSs) of Pitx1 and Pitx2 were downloaded from NCBI GeneBank. Pitx1 and Pitx2 coding sequences of *Ancistrus sp.* were found from our de novo RNA-sequencing data. Multiple amino acid sequence alignments were conducted using MUSCLE via Molecular Evolutionary Genetics Analysis X (MEGAX, https://www.megasoftware.net/). The phylogenetic tree of amino acid sequences was established by using the maximum likelihood method with 500 bootstrap replicates to estimate confidence values of phylogenetic tree (MEGAX).

## Supplementary Information

Supplementary Information.

## Data Availability

De novo RNA sequencing assembly data have been deposited in the National Center for Biotechnology Information Sequence Read Archives (NCBI SRA), www.ncbi.nlm.nih.gov/sra (BioProject accession code PRJNA288370).
