# Supplementary material for: Redeployment of odontode gene regulatory network underlies dermal denticle formation and evolution in suckermouth armored catfish
Source: Sci Rep. 2022 Apr 13;12:6172. doi: 10.1038/s41598-022-10222-y (PMC9007992; doi:10.1038/s41598-022-10222-y)
Supplement: Supplementary file 1 — Supplementary Information. [file 41598_2022_10222_MOESM1_ESM.pdf]

Supplementary Material

Redeployment of odontode gene regulatory network underlies dermal denticle formation and evolution in suckermouth armored catfish

Shunsuke Mori[a*] and Tetsuya Nakamura[a*]

Authors' affiliations

[a] Department of Genetics, Rutgers the State University of New Jersey, Piscataway, NJ, 08854, USA

[*] Address correspondence to: Shunsuke Mori (no0423@gmail.com) and Tetsuya Nakamura (nakamura@dls.rutgers.edu)

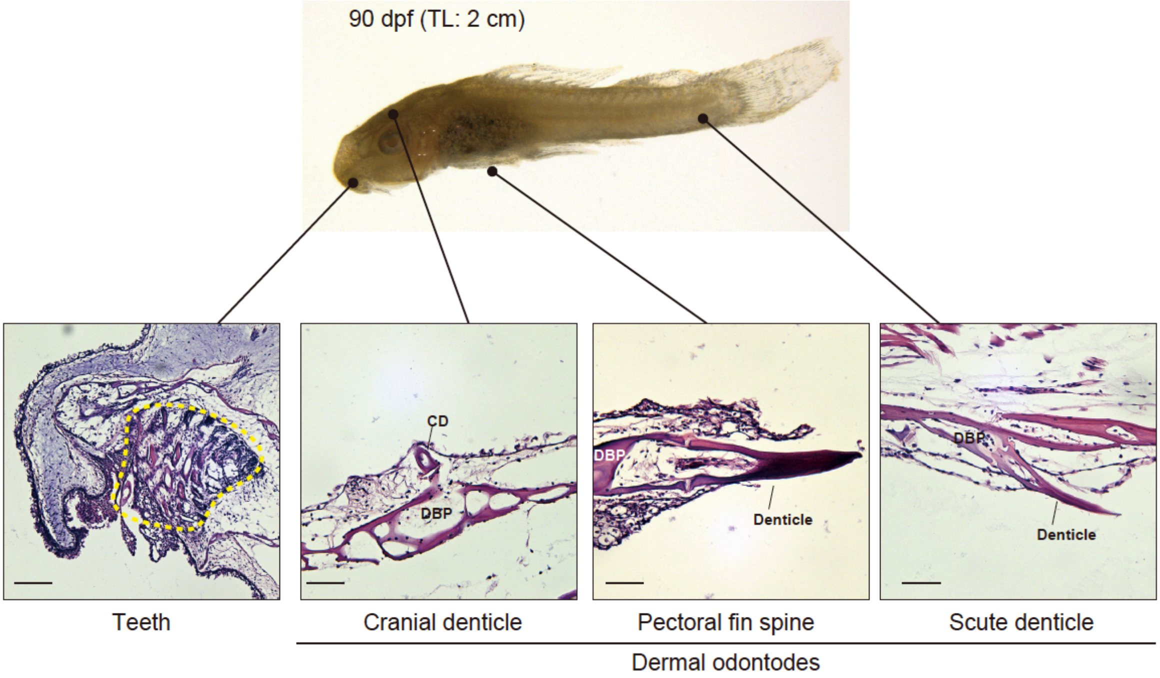

Fig. S1. Formation of dermal odontodes in *Ancistrus sp.*.

Representative pictures of *Ancsitrus sp.* juvenile at 90 dpf (TL: 2 cm, top) and HE-stained sagittal sections of oral tissue, cranial dermal denticles, pectoral fin spines, and scute denticles (bottom panels). Two biological replicates were investigated in this experiment. The yellow dotted area indicates teeth. Scale bar: 0.5 mm. CD; cranial dermal denticle, SB; skull bone, DBP; dermal bony plate.

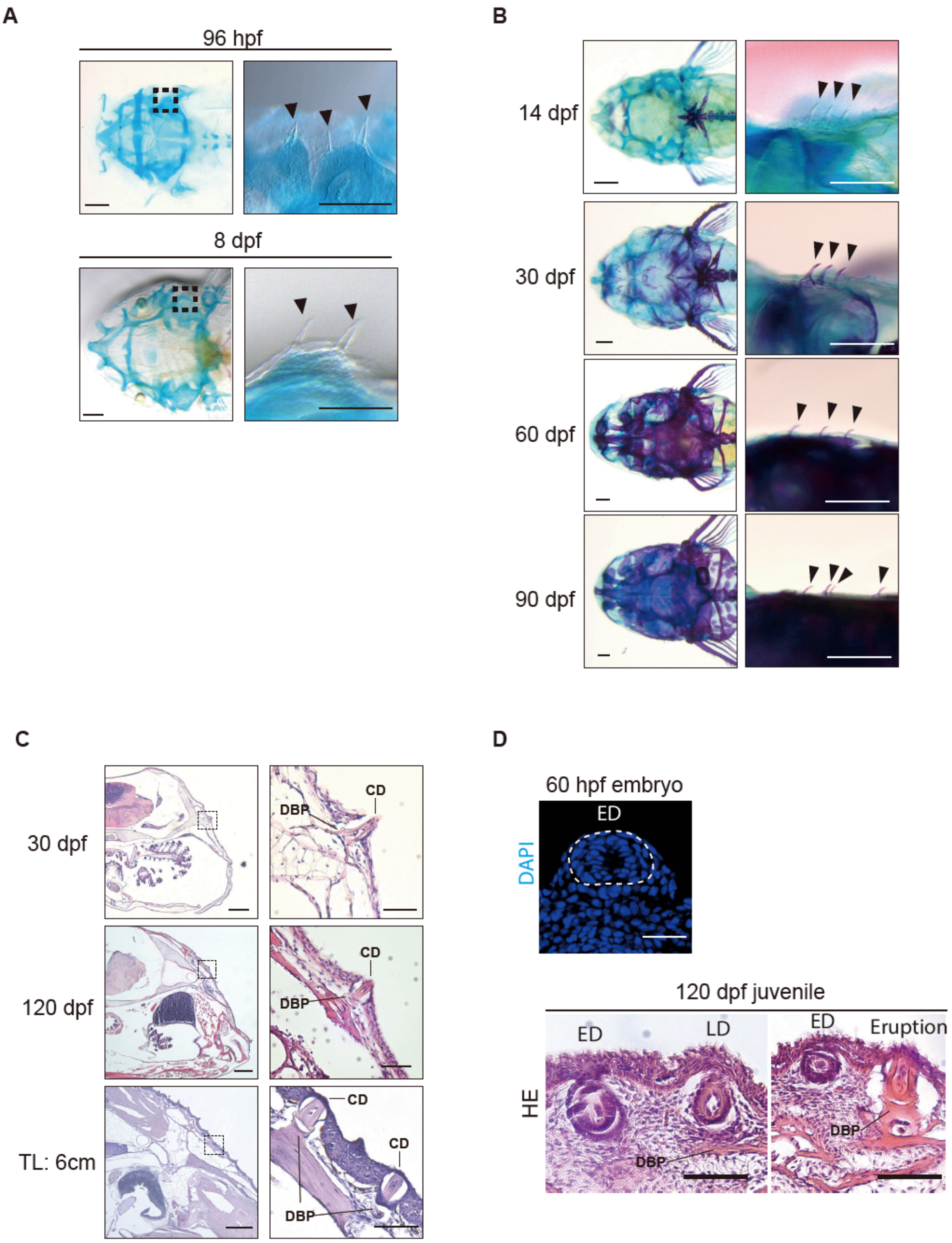

Fig. S2. Whole-mount and histological analysis of developing cranial dermal denticles from the embryonic to the adult stage.

(A) Bone and cartilage staining of the developmental series of suckermouth armored catfish embryos at 96 hpf (left) and larva at 8 dpf (right). Each right panel shows higher magnification of the dotted box area in the left panels. Arrowheads indicate the primary cranial dermal denticles. Scale bars: 0.5 mm. (B) Bone and cartilage staining of the

developmental series of juveniles at 14, 30, 60, and 90 dpf. Each right picture shows high-magnification view of the secondary cranial dermal denticle formation sites (arrowheads) in the left pictures. Scale bar: 0.5 mm. (C) HE-stained head transverse sections of the juveniles at 30 dpf, 120 dpf, and the early adult stage (TL: 6 cm). Each right panel shows high-magnification view of the dotted box area in the left panels. Scale bars: 0.5 mm (left) and 0.1 mm (right). (D) Nuclear staining (DAPI) of a transverse section of a primary cranial dermal denticle at the early differentiation stage (ED) in 60 hpf embryo (top panel). HE-staining of transverse sections of 120 dpf juvenile (TL: 3 cm, bottom panels). Secondary cranial dermal denticles form in the cranial epidermis through the ED, late differentiation (LD), and eruption stages. Scale bars: 100 µm. CD; cranial dermal denticle, DBP; dermal bony plate. At least two biological replicates were investigated in each experiment.

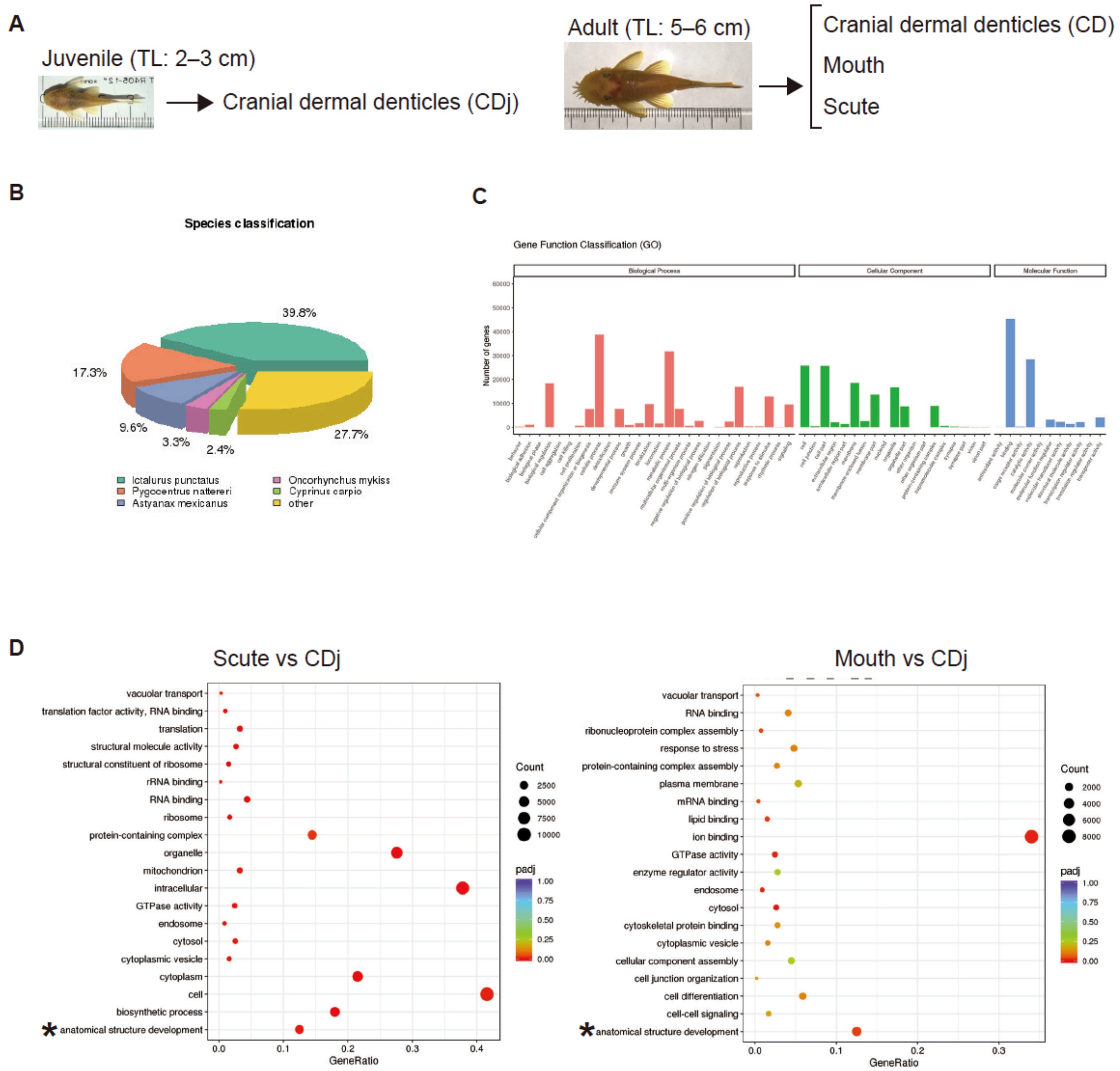
A
Juvenile (TL: 2–3 cm)
Cranial dermal denticles (CDj)
Adult (TL: 5–6 cm)
Cranial dermal denticles (CD)
Mouth
Scute
B
Species classification
39.8%
17.3%
9.6%
3.3%
2.4%
27.7%
Ictalurus punctatus
Pygocentrus nattereri
Astyanax mexicanus
Oncorhynchus mykiss
Cyprinus carpio
other
C
Gene Function Classification (GO)
Biological Process
Cellular Component
Molecular Function
Number of genes
D
Scute vs CDj
vacuolar transport
translation factor activity, RNA binding
translation
structural molecule activity
structural constituent of ribosome
rRNA binding
RNA binding
ribosome
protein-containing complex
organelle
mitochondrion
intracellular
GTPase activity
endosome
cytosol
cytoplasmic vesicle
cytoplasm
cell
biosynthetic process
anatomical structure development
GeneRatio
Count
padj
Mouth vs CDj
vacuolar transport
RNA binding
ribonucleoprotein complex assembly
response to stress
protein-containing complex assembly
plasma membrane
mRNA binding
lipid binding
ion binding
GTPase activity
enzyme regulator activity
endosome
cytosol
cytoskeletal protein binding
cytoplasmic vesicle
cellular component assembly
cell junction organization
cell differentiation
cell-cell signaling
anatomical structure development
GeneRatio
Count
padj

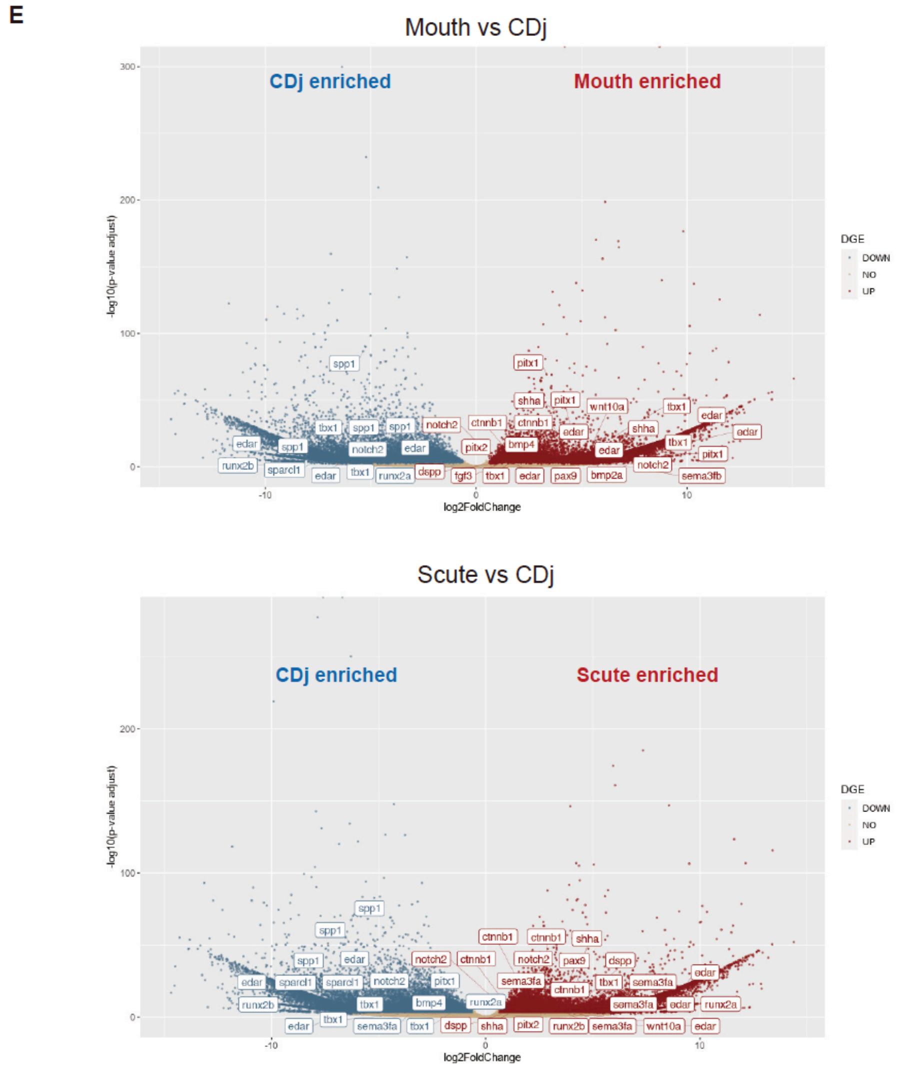

Fig. S3. *De novo* transcriptome profiling in the odontode tissues of *Ancistrus sp.*.

(A) *De novo* RNA-seq was performed by using odontode tissues collected from juvenile cranial skins (left, TL: 2–3 cm, *n*=3) and adult cranial skins, mouths, and scutes (right, TL: 5–6 cm, *n*=3). (B) Top-hit species distribution based on Nr-annotated unigenes. (C) Functional gene classification of GO-annotated unigenes. (D) GO enrichment analysis of the enriched genes in mouths (left) and scutes (right), compared to CDj. The vertical axis represents the GO terms and the horizontal axis does the enrichment of each term. The size of each point represents the number of upregulated genes in the GO terms, and the color of the point represents the adjusted *p*-value. (E) The volcano plots display the enriched genes in Mouth (top) and Scute (bottom) relative to CDj. Significantly enriched genes in CD are highlighted

in red (log2foldchange > 0.6 and adjusted *p*-value < 0.01) and enriched genes in CDj are in blue (log2foldchange < -0.6 and adjusted *p*-value < 0.01). The gene symbols show differentially expressed genes associated with oGRN and odontogenesis. As different unigenes were annotated by the same gene names or homologous genes in different species, the multiple same gene names were shown in the plot.

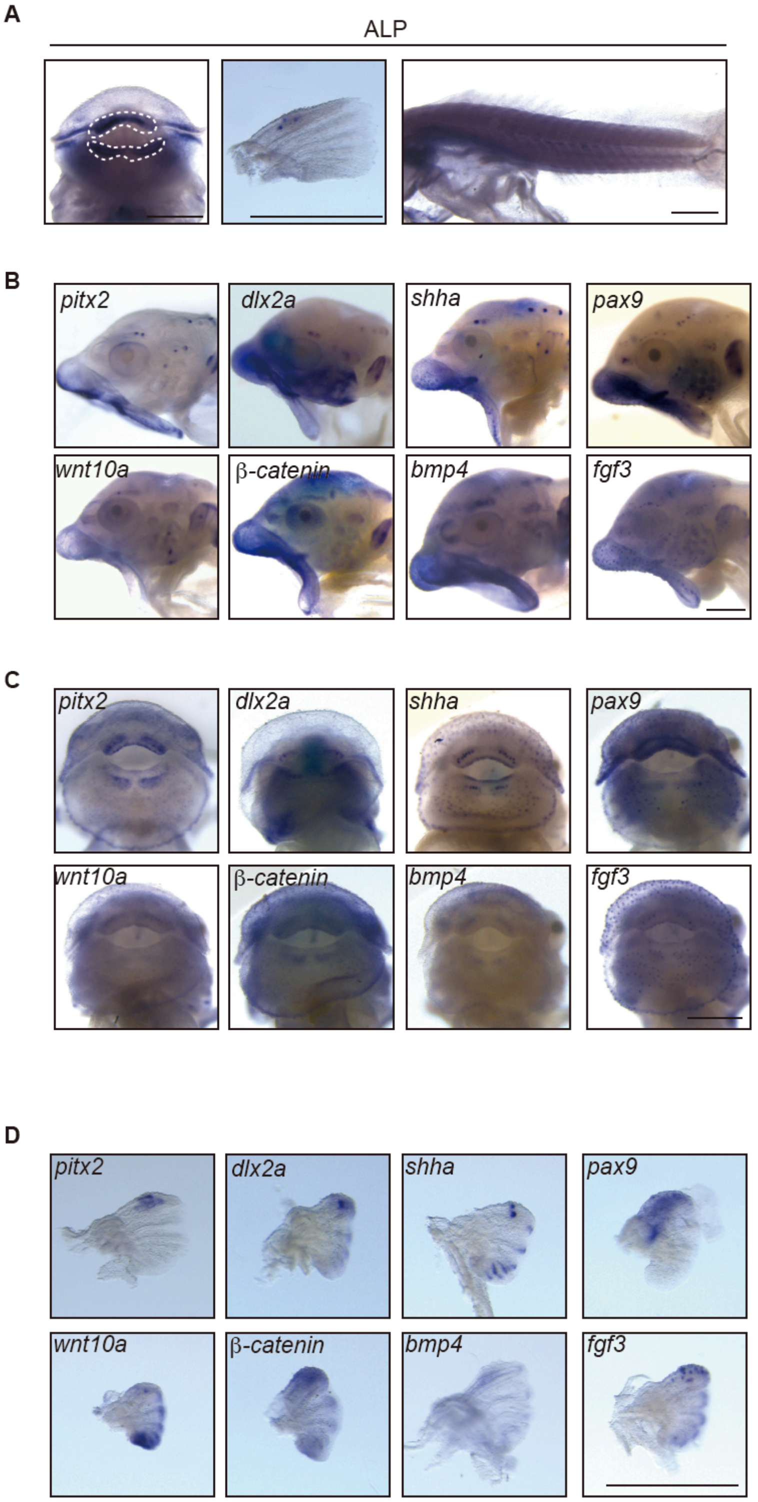

Fig. S4. Expression of oGRN genes in the odontode tissues of *Ancsitrus sp.*.

(A) ALP staining of teeth (left, white dotted outline), pectoral fins (middle), and the trunk (right) of *Ancistrus sp.* embryos at 96 hpf. Teeth and pectoral fin spines, not the trunk, showed restricted ALP staining. (B–D) Whole-mount *in situ* hybridization of *pitx2, dlx2a,*

shha, pax9, wnt10a, b-catenin, bmp4, and *fgf3*. All of these gene transcripts were detected in the cranial dermal denticles (B), teeth (C), and pectoral fin spines (D). Scale bars: 0.5 mm. At least three biological replicates were investigated in each experiment.

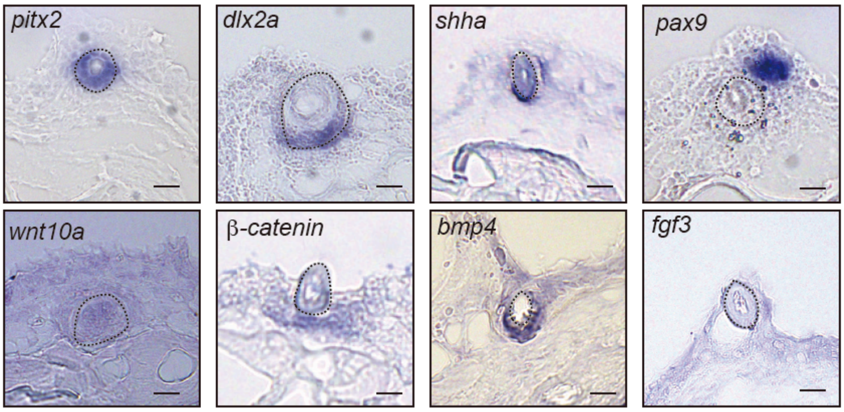

Fig. S5. Expression of oGRN genes in the secondary cranial dermal denticles.

Transverse sections of whole-mount *in situ* hybridization of *pitx2, dlx2a, shha, pax9, wnt10a, b-catenin, bmp4,* and *fgf3* in juvenile (120 dpf, TL: 3 cm) cranial dermal denticles. Scale bars: 100 µm. Dotted circles indicate denticle germ. Note that, to display the representative oGRN gene expression patterns, we selected different slice positions of dermal denticles for some of the section ISH photos. Accordingly, some denticle germ morphology appear to be different from others. These data were obtained from at least two biological replicates.

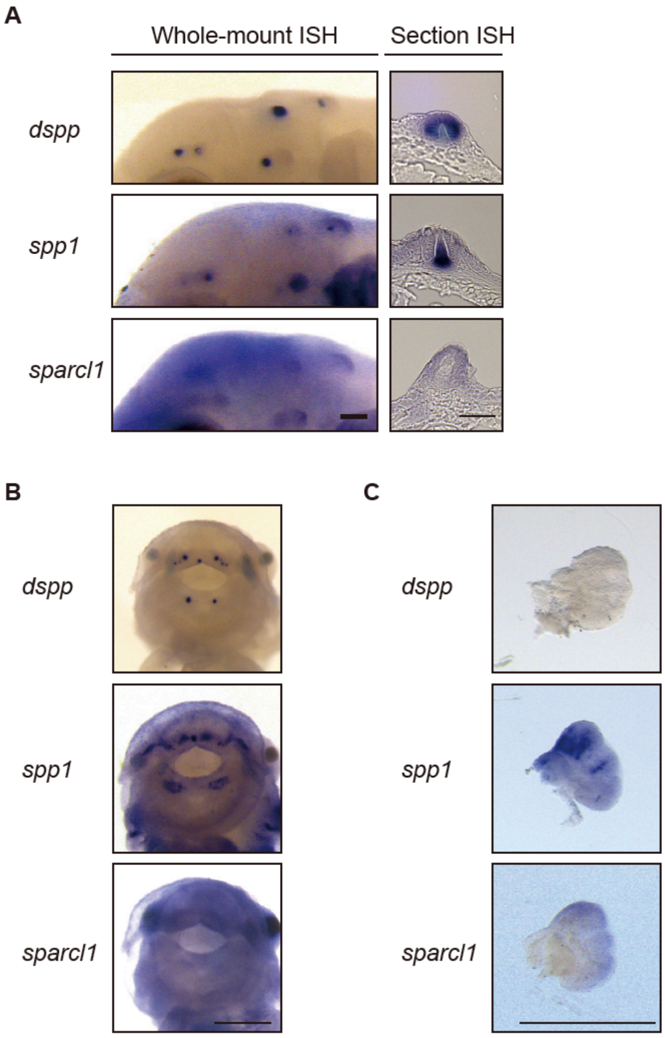

Fig. S6. Expression of dentinogenesis-related genes in the primarily cranial dermal denticles.

(A-C) Whole-mount *in situ* hybridization of *dspp, spp1,* and *sparcl1*. These gene transcripts were detected in the cranial dermal denticles (A), teeth (B). While pectoral fin spines expressed both *spp1* and *sparcl1*, but not *dspp.* (C). Scale bars: 0.5 mm. At least three biological replicates were investigated in each experiment.

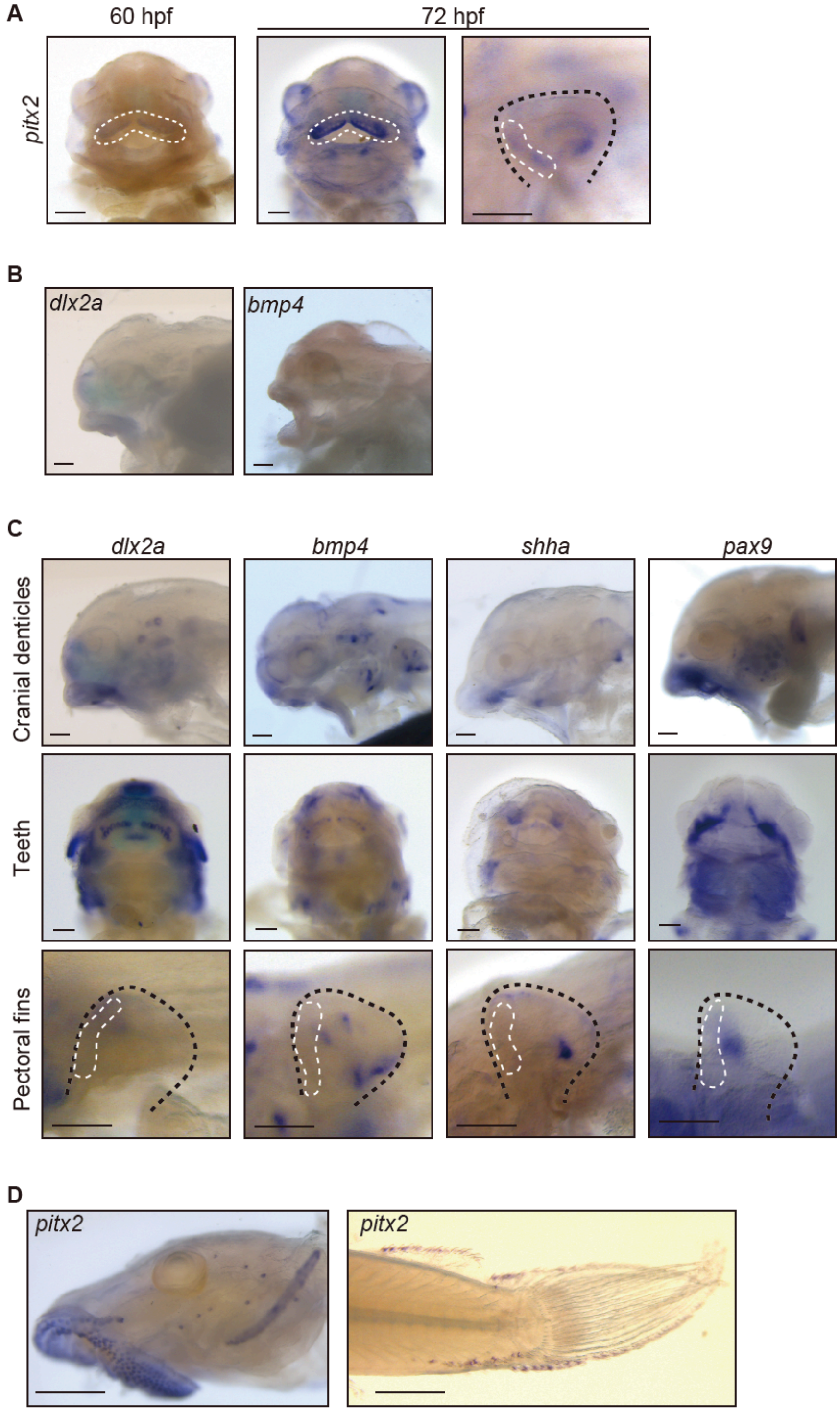

Fig. S7. *Pitx2* is expressed at the initiation stage of cranial dermal denticle development. (A) Whole-mount *in situ* hybridization of *pitx2* in *Ancistrus sp.* embryo at 60 hpf (teeth) and 72 hpf (left: teeth and right: pectoral fin, black dotted outline). The expression of *pitx2* was initiated in teeth (white dotted circles) and pectoral fin spines (white dotted circle) from 60 hpf and 72 hpf, respectively. Scale bars: 100 µm. (B) Whole-mount *in situ* hybridization of

dlx2a and *bmp4* in *Ancistrus sp.* at 60 hpf. *Dlx2a* and *bmp4* transcripts were not detected. Scale bars: 100 µm. (C) Whole-mount *in situ* hybridization of *dlx2a, bmp4, shha,* and *pax9* in cranial dermal denticles (top), teeth (middle), and pectoral fin spines (bottom, black dotted outline) of *Ancistrus sp.* embryos at 72 hpf. Scale bars: 100 µm. *Dlx2a* and *bmp4* but not *shha* and *pax9* transcripts were detected in the cranial dermal denticles and teeth germs at 72 hpf. At this stage, all of these genes are not expressed at the prospective spine denticle sites (white dotted circles). (D) Whole-mount *in situ* hybridization of *pitx2* in *Ancistrus sp.* juvenile at 30 dpf. The expression of *pitx2* was detected at newly forming cranial dermal denticles in the head (left) and the trunk (right). Scale bars: 0.5 mm. At least three biological replicates were investigated in each experiment.

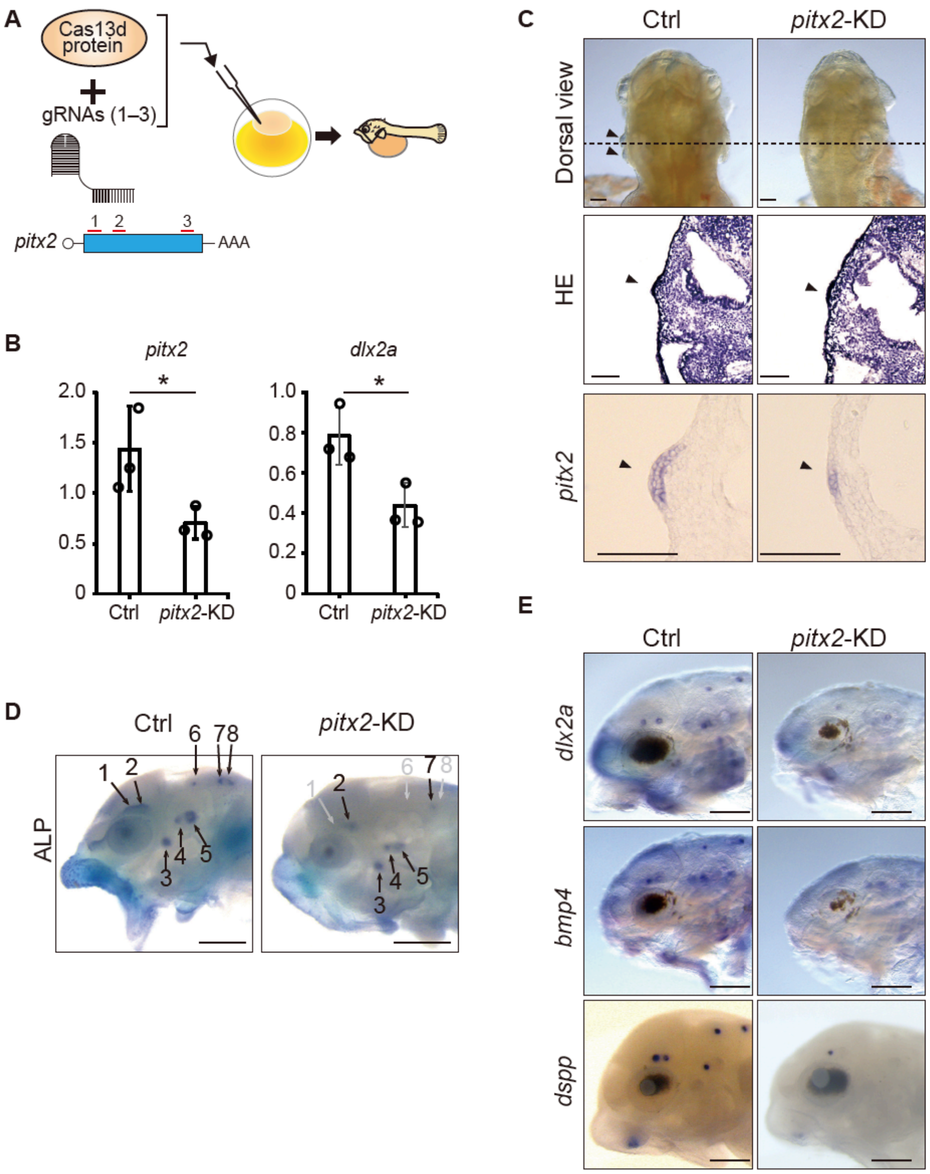

Fig. S8. Pitx2 is an inducer of cranial dermal denticle placodes.

(A) Schematic diagram of *pitx2* knockdown method for *Ancistrus sp.* embryos using CRISPR-RfxCas13d. Three gRNA targeting sites in *pitx2* coding sequence are shown by red lines. RfxCas13d protein was mixed with/without the gRNAs (*pitx2*-KD or Ctrl, respectively) and injected into one- to two-cell stage *Ancistrus sp.* embryos. (B) Quantitative RT-PCR analysis of *pitx2* and *dlx2a* expression level in Ctrl and *pitx2*-KD embryos at 72 hpf (mean ±s.d, n=3, *$P < 0.05$; Wilcoxon signed rank test). (C) Representative gross photos of Ctrl (top, left) and *pitx2*-KD (top, right) embryos at 60 hpf. HE staining (middle panels) and *in situ* hybridization of *pitx2* (bottom panels) were conducted using transverse sections at the

positions of the dotted lines in top panels. Arrowheads indicate the epithelial placodes. Note that the placode did not thicken in *pitx2*-KD embryos, and *pitx2* expression was downregulated. Scale bars: 100 µm. (D) ALP staining of Ctrl (left) and *pitx2*-KD (right) embryos at 96 hpf. Each numbering position of cranial dermal denticle indicates the corresponding site between Ctrl and *pitx2*-KD embryos. Gray-colored numberings and arrows indicate the defected cranial dermal denticles. Scale bars: 0.5 mm. (E) Whole-mount *in situ* hybridization of *dlx2a* (top) and *bmp4* (bottom) in Ctrl and *pitx2*-KD embryos at 96 hpf. Scale bars: 0.5 mm. At least three biological replicates were investigated in each experiment.

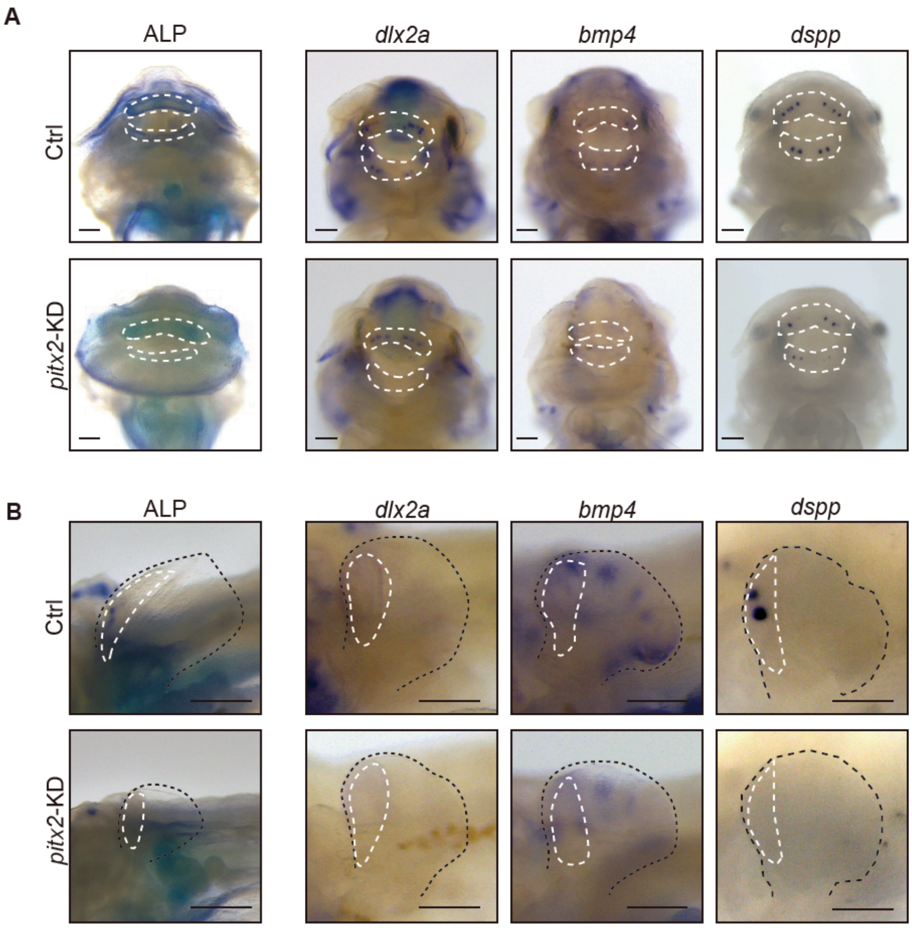

Fig. S9. Knockdown of *pitx2* reduces teeth and pectoral fin spines formation.

(A, B) Embryos injected with only Cas13d protein (Ctrl)- or Cas13d protein with *pitx2*-

gRNAs (*pitx2*-KD) at 96 hpf. Knockdown of *pitx2* reduced the ALP staining and the expression of *dlx2a, bmp4* and *dspp* in teeth (A, white dotted circles, scale bars: 0.5 mm) and pectoral fin spines (B, white dotted circles, Scale bars: 100 µm.). Dotted black outlines indicate the pectoral fins in B. At least three biological replicates were investigated in each experiment.

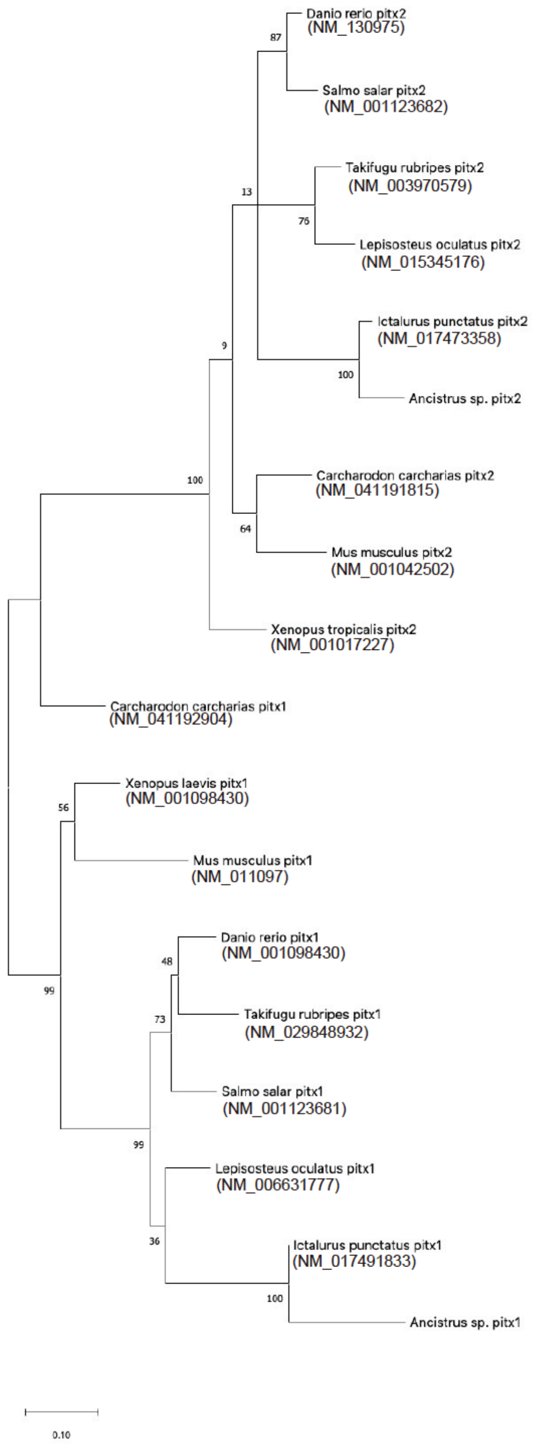

Fig. S10. Evolutionary divergence of pitx1 and pitx2 amino acid sequences across vertebrate species.

Phylogenetic analysis by maximum likelihood method based on amino acid sequences of pitx1 and pitx2 of mouse (*Mus musculus*), frog (*Xenopus laevis*), great white shark (*Carcharodon carcharias*), gar (*Lepisosteus oculatus*), Salmon (*Salmon salar*), fugu (*Takifugu rubripes*), zebrafish (*Danio rerio*), channel catfish (*Icalurus punctatus*), and suckermouth armored catfish (*Ancistrus sp.*). GeneBank Accession numbers used in this analysis are given in the brackets under each species name. Bootstrap values from 500 replicate trees are indicated at each node. Scale bar shows amino acid sequence divergence.

Table. S1. Viability after injection of RfxCas13d with/without *pitx2*-gRNA .

**Control (Cas13d protein only)**	Experiment_1	Experiment_2	Experiment_3	Average
Viability (at 48 hpf)	1/5 (20.0%)	4/10 (40.0%)	6/10 (60.0%)	40.0% (± 16.3)
Viability (at 96 hpf)	1/1 (100%)	4/4 (100%)	5/6 (83.3%)	94.4% (± 7.9)
**Pitx2 gRNA + Cas13d protein**	Experiment_1	Experiment_2	Experiment_3	Average
Viability (at 48 hpf)	12/50 (24.0%)	22/50 (44.0%)	20/50 (40.0%)	36.0% (±8.6)
Viability (at 96 hpf)	4/12 (33.3%)	7/22 (31.8%)	7/20 (35.0%)	33.4% (±1.3)

*Each experiment column shows the number and rate of survival embryos out of all embryos used for each experiment. Note that the total number of 96 hpf embryos is the equal number of survived ones at 48 hpf.

Table. S2. The number of *dspp*-positive dermal denticles and teeth in *pitx2* knockdown embryos.

	**Cranial dermal denticles**	**Teeth**	**Spines**
**Control (Cas13d protein only)_1**	8/8	10/10	4/4
**Control (Cas13d protein only)_2**	8/8	10/10	1/4
**Control (Cas13d protein only)_3**	8/8	10/10	2/4
**Pitx2 gRNA + Cas13d protein_1**	1/8	8/10	0/4
**Pitx2 gRNA + Cas13d protein_2**	1/8	0/10	0/4
**Pitx2 gRNA + Cas13d protein_3**	3/8	8/10	0/4
**Pitx2 gRNA + Cas13d protein_4**	3/8	6/10	1/4
**Pitx2 gRNA + Cas13d protein_5**	6/8	7/10	0/4

Table. S3. RfxCas13d oligonucleotides and primers list.

CRISPR-Cas13d	Name	Sequence (5'-3')
	Cas13d_Universal_t7	TAATACGACTCACTATAGGAACCCCTACCAACTGGTCGGGGTTTGAAAC
	Cas13d_pitx2_1	AGCTTGTGTCAACGTGTGTGCAGTTTCAAACCCCGACCAGTT
	Cas13d_pitx2_2	TGGACGAACCTCACGGAGGCGCGTTTCAAACCCCGACCAGTT
	Cas13d_pitx2_3	TGCGTGTCCTTACGCCCCGCCAGTTTCAAACCCCGACCAGTT
	Cas13d_pitx2_4	CTTTGTCCTCGCAAGCCATGTTGTTTCAAACCCCGACCAGTT
	Cas13d_pitx2_5	CGCAGTTCAACGGCCTGGTGCAGTTTCAAACCCCGACCAGTT
	Cas13d_pitx2_6	GTGCAGAGTAAAAGTGAAGACTGTTTCAAACCCCGACCAGTT

ISH cloning primers	Gene name	Forward (5'-3')	Reverse (5'-3')
	pitx2	ATGGATTCCCACTGCCGAAA	TTAAACTGGTCTGTCCACGG
	shha	AAGCACCCGAAGAAGCTGGC	TCAGCTCTGCTCCTGCATGC
	bmp4	ATGATTCCTGGTAATCGAAT	CTAGCGACAGCCACACCCCT
	fgf3	ATGCTCGTAGTTCAGCTCTT	TTACTTTTTGGCCAGTCTGG
	pax9	ATGGAACCAGCCTTTGGGGA	TCACAGCTGTGGGGAGAGAG
	wnt10a	CCAAAGACTTCCTGGACTCG	TTGCAGACACTGACCCACTC
	ctnnb1	TGCACATTCTTGCCAGAGAC	ATCAAACCAAGCGAGCTGAT
	dlx2a	ATGACTGGGGTGTTTGACAG	TCAAAATATCGTCCCGGCGC

qRT-PCR	Gene name	Forward (5'-3')	Reverse (5'-3')
	pitx2	AGAGGACGCACTTCACCAGT	GCGACGGTTCTTAAACCAAA
	actb1	AACACCCCAGCCATGTATGT	GGCAGGGCATAACCTTCATA
	dlx2a	GCCGTTCGAAGTTCAAGAAG	AAAGCCGAGCTCACACTGTT
